# Moisture-responsive root-branching pathways identified in diverse maize breeding germplasm

**DOI:** 10.1126/science.ads5999

**Published:** 2025-02-06

**Authors:** Johannes D. Scharwies, Taylor Clarke, Zihao Zheng, Andrea Dinneny, Siri Birkeland, Margaretha A. Veltman, Craig J. Sturrock, Jason Banda, Héctor H. Torres-Martínez, Willian G. Viana, Ria Khare, Joseph Kieber, Bipin K. Pandey, Malcolm Bennett, Patrick S. Schnable, José R. Dinneny

**Affiliations:** 1 Department of Biology, Stanford University; Stanford, CA 94305, USA.; 2 Howard Hughes Medical Institute, Stanford University; Stanford, CA 94305, USA.; 3 Department of Agronomy, Iowa State University; Ames, IA 50011-1085, USA.; 4 Faculty of Chemistry, Biotechnology and Food Science, Norwegian University of Life Sciences; Ås, 1432, Norway.; 5 Natural History Museum, University of Oslo; Oslo, 0318, Norway.; 6 Plant and Crop Sciences, School of Biosciences, University of Nottingham; Sutton Bonington, LE12 5RD, UK.; 7 Department of Biology, University of North Carolina; Chapel Hill, NC 27599, USA.

## Abstract

Plants grow complex root systems to extract unevenly distributed resources from soils. Spatial differences in soil moisture are perceived by root tips leading to the patterning of new root branches towards available water, a process called hydropatterning. Little is known about hydropatterning behavior and its genetic basis in crops plants. Here, we develop an assay to measure hydropatterning in maize and reveal substantial differences between tropical/subtropical and temperate maize breeding germplasm that likely resulted from divergent selection. Genetic dissection of hydropatterning confirmed the regulatory role of auxin and revealed that the gaseous hormone ethylene locally inhibits root branching from air-exposed tissues. Our results demonstrate how distinct signaling pathways translate spatial patterns of water availability to developmental programs that determine root architecture.

Climate change is predicted to increase the duration and severity of droughts (1). This will threaten crop production, which depends heavily on water. Plant water uptake is facilitated by an intricate network of roots. Breeding plants with improved root access to water is a potential method to make crops resilient to climate change (2). Root networks are established by branching of the primary root axis. The development of lateral root branches is highly responsive to the spatio-temporal distribution of resources like water and nutrients in soils (3, 4). Plants sense micron-scale heterogeneity in water availability at their root tips with spatial differences along the root-tip circumference determining the patterning of lateral roots through hydropatterning (5, 6) (Fig. 1A). This response may allow plants to capture water more efficiently while minimizing the metabolic cost of root growth in dry soil (7). Understanding the extent of phenotypic variation for this trait within breeding populations and determining its genetic basis may facilitate crop improvement. Furthermore, understanding the mechanistic basis of hydropatterning illuminates how heterogeneity in moisture is sensed by organisms to enact an adaptive response.

## Hydropatterning in domesticated maize

**Fig. 1.**
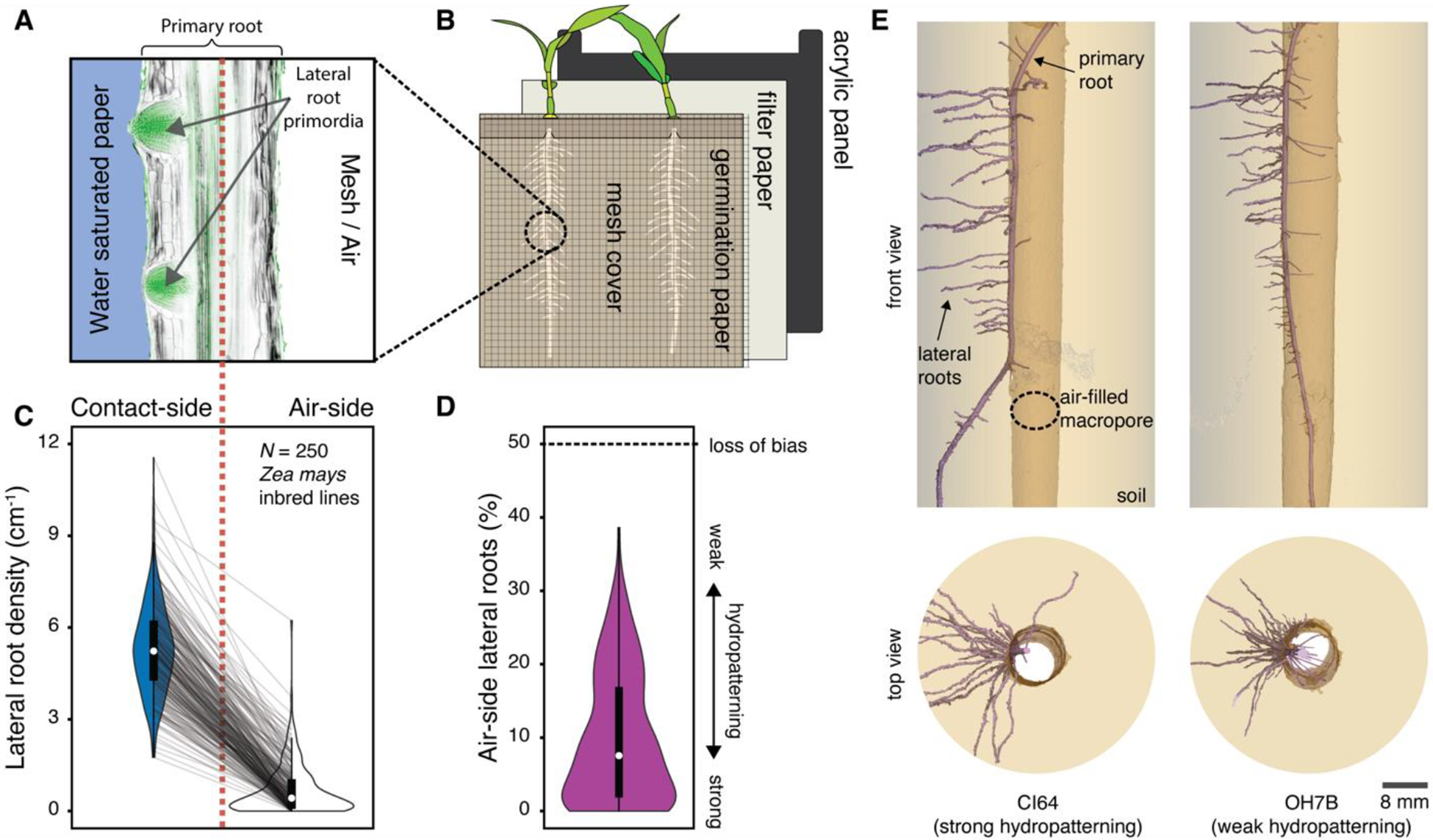
Hydropatterning responses revealed in public sector breeding lines of *Zea mays* (maize). (**A and B**) Schematic of the (A) hydropatterning response in (B) our custom-built hydropatterning assay. Primary roots of maize seedlings are grown in a vertical position along moist paper while being prevented from growing off the paper by a mesh cover. Lateral root primordia are preferentially induced towards the water-saturated paper (contact-side) and suppressed on the air-exposed side (air-side). Longitudinal cross-section of maize root (B73 inbred) stained with Calcofluor White (cell walls; gray) and SYBR Green (lateral root primordia; green). Contact- and air-side are separated by a dashed red line. (**C and D**) Distribution of (C) contact- (blue) and air-side (white) lateral root densities from 250 maize inbred lines characterized using the hydropatterning assay and calculated (D) percent air-side lateral roots (purple). Each inbred line is represented by its median value (*n* = 1 – 3 seedlings/inbred) (data S2). Gray lines connect corresponding inbred lines. Population median (white circles). (**E**) 3D rendered X-ray Computed Tomography images, viewed from the front and the top, showing lateral root patterning on primary roots of strong (CI64) and weak (OH7B) hydropatterning inbred lines grown through an air-filled macropore in soil.

Here we investigated hydropatterning in the cereal crop species *Zea mays* (maize), which constitutes a major source of calories worldwide. To capture the phenotypic diversity of hydropatterning, we developed an assay using germination paper to create a controlled gradient of water availability across the circumference of the growing primary root (Fig. 1B and fig. S1). Simultaneous characterization of a diverse set of 250 maize inbred lines (data S1) from the Goodman-Buckler association panel allowed us to cover the majority of genetic diversity present in current public-sector breeding programs (8).

For all tested maize inbred lines, we observed that primary roots preferentially formed lateral roots on the side touching the water-saturated germination-paper (contact-side), which is consistent with the inductive effect of water availability previously observed in the maize reference inbred B73 and in other species (5) (Fig. 1C, data S2). Nevertheless, a substantial proportion of all surveyed maize inbred lines developed air-side lateral roots as well, resulting in an observed phenotypic range of 0 – 39% air-side lateral roots across all 250 inbred lines (Fig. 1D). This suggests that a larger portion of maize inbred lines exhibit weakened hydropatterning (more air-side lateral roots) than previously indicated (9). Additionally, we observed in a set of 20 maize inbred lines with diverse hydropatterning responses in primary roots, that hydropatterning in crown roots, which make up the bulk of mature maize root systems, is significantly correlated (fig. S2, A to C and data S3). Our results highlight the substantial variation of hydropatterning across maize root types, allowing this trait to be used for understanding how quantitative genetic variation contributes to overall root architecture.

In the B73 maize inbred line, lateral root founder cells are initiated around 12 mm from the root tip (10). Prior research using B73 found that moisture availability cues, which determine the patterning of lateral roots, are perceived closer to the root tip, specifically within the first 5–6 mm (9). This suggests that moisture cues act on lateral root development at the founder cell patterning stage rather than at later developmental stages. Comparing lateral root patterning in several strong (< 5% air-side lateral roots) and weak (> 20% air-side lateral roots) hydropatterning inbred lines, we found that pre-emerged lateral root primordia and post-emergence lateral roots exhibited the same bias in distribution between contact- and air-sides corresponding to the hydropatterning strength of each inbred line (fig. S2, D and E). Our observations provide further evidence that hydropatterning primarily acts at the lateral root founder cell patterning stage.

We next examined how the observed variation in hydropatterning correlates with root architecture in soil conditions. In nature, large air-spaces in the soil matrix, called macropores, are commonly created by prior root growth or burrowing invertebrate activity, such as by earthworms. We tested how lateral roots were patterned when primary roots were grown through artificial macropores. Strong hydropatterning inbred lines initiated their lateral roots preferentially towards the side of the root in contact with soil, as observed by microscale X-ray Computed Tomography (Fig. 1E). In contrast, weak hydropatterning inbred lines displayed a reduced bias with more lateral roots growing into the air-filled macropore. Quantification across multiple strong and weak hydropatterning inbred lines found that they made similar percentages of air-side lateral roots in the soil macropore and our hydropatterning assay (fig. S2, F to H). The results demonstrate that our hydropatterning assay generates reproducible phenotypes that translate to soil conditions.

To explore how variation in hydropatterning relates to other phenotypic traits of field-grown maize plants, we performed a correlation analysis with 64 compiled trait sets measured from field-grown plants (11). We found that both air-side lateral root density and percent air-side lateral roots correlate significantly with root crown depth and the number of nodes with brace roots (fig. S3, A to C and data S4). Weaker hydropatterning inbred lines generally exhibited more shallow root systems with fewer brace roots according to data collected by two studies from Iowa (fig. S3, D to F) (12, 13). This suggests that more efficient placement of root branches towards water may improve the ability of root systems to attain greater depths, possibly due to the metabolic savings achieved by limiting branching in dry soil (14). Importantly, no significant correlations were observed with contact-side lateral root density, suggesting that this trait has less relevance to the in-field root architecture traits measured.

## Variation and selection of hydropatterning across maize breeding subpopulations

**Fig. 2.**
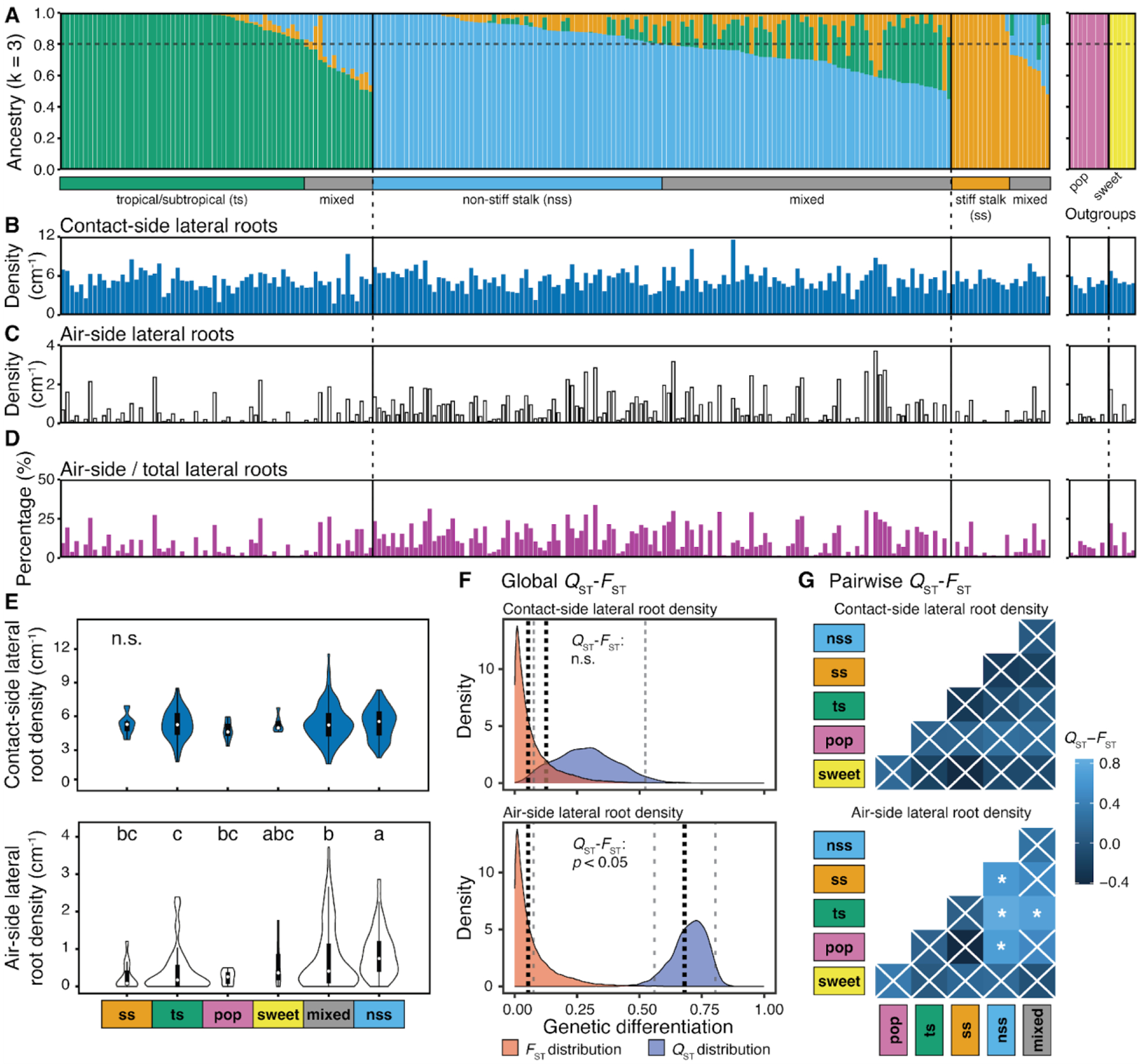
Differences in hydropatterning across breeding subpopulations may have been caused by divergent selection. (**A - D**) Population structure and hydropatterning traits of 231 maize inbred lines (19 inbred lines from original population of *N* = 250 were excluded due to missing genotypic data). (A) Ancestry components and subpopulation assignments. Inbred lines < 80% group identity = “mixed”. Popcorn (pop) and sweet corn (sweet) groups were defined a priori. Phenotypic data of (B) contact-side and (C) air-side lateral root density, and (D) percent air-side lateral roots are shown as the median value for each inbred line (*n* = 1 – 3 seedlings/inbred) (data S2). (**E**) Subpopulation comparisons of contact-side (top) and air-side (bottom) lateral root density. Violin plot area adjusted for number of inbred lines/subpopulation. Letters denote significant differences between subpopulations (*p* ≤ 0.05, Kruskal–Wallis and Dunn’s post hoc tests); n.s., no significant differences. (**F**) Population-wide comparison of *F*_ST_ (fixation index) and *Q*_ST_ (genetic differentiation regarding a quantitative trait) distributions for contact-side (top) and air-side (bottom) lateral root density. Black dotted lines (means), gray dashed lines (95% confidence intervals). (**G**) Subpopulation pairwise *Q*_ST_ - *F*_ST_ comparisons. Asterisks denote significant differences between *Q*_ST_ and *F*_ST_ (Benjamini & Hochberg-adjusted: * *p* ≤ 0.05); white crosses, not significant. Number of inbred lines in each subpopulation across all panels: *n*_ts_ = 53, *n*_nss_ = 63, *n*_ss_ = 14, *n*_mixed_ = 88, *n*_pop_ = 9, *n*_sweet_ = 6.

We reassessed population structure and assigned subpopulations for all phenotyped inbred lines that had matching whole-genome sequencing data available (*n* = 231). Inbred lines with less than 80% subpopulation identity were assigned to a mixed group (8) (Fig. 2A and data S5). While variation in contact-side lateral root density was uniform across all subpopulations, inbred lines with higher air-side lateral root density and percent air-side lateral roots were predominantly associated with the non-stiff stalk/mixed groups (Fig. 2, B to D). This led to significantly weaker hydropatterning for the large temperate non-stiff stalk group compared to the large tropical/subtropical group or the smaller temperate stiff stalk group (Fig. 2E and fig. S4A).

To test whether this divergence in hydropatterning was best explained by neutral evolution or selection, we compared quantitative genetic differentiation with regards to contact- or air-side lateral root density (*Q*_ST_) to the population genetic differentiation due to genetic structure (*F*_ST_). We found that *Q*_ST_ and *F*_ST_ distributions overlapped for contact-side lateral root density, which suggests that genetic differentiation for this trait occurred through neutral evolution. Conversely, *Q*_ST_ was significantly in excess of *F*_ST_ for air-side lateral root density, as well as percent air-side lateral roots, indicating divergence through differential selection for these hydropatterning traits (Fig. 2F, fig. S4B, and data S6). Pairwise comparisons between subpopulations show the largest *Q*_ST_ - *F*_ST_ differences between the non-stiff stalk and tropical/subtropical groups, suggesting that divergence in selective pressures for hydropatterning occurred predominantly after the split between tropical/subtropical and temperate germplasm (Fig. 2G, fig. S4C, and data S6). Stronger hydropatterning in the tropical/subtropical subpopulation may have resulted from selection for drought tolerance, among other abiotic stress factors, during breeding (15, 16). In contrast, weakened hydropatterning in the temperate non-stiff stalk subpopulation could be the result of relaxation in selection pressures on efficient water uptake in temperate environments. However, linkage between hydropatterning and other traits could have contributed to differences in hydropatterning between subpopulations. Pronounced *Q*_ST_ > *F*_ST_ differences for air-side lateral root density and contrasting observations for contact-side lateral root density support our hypothesis but inbreeding may inflate *Q*_ST_ estimates (17).

## Genetic architecture of hydropatterning in domesticated maize

Previous work in *Arabidopsis thaliana* (Arabidopsis) has shown that the auxin hormone signaling pathway promotes branching on the contact-side of roots during hydropatterning. Mutants that disrupt auxin biosynthesis and polar transport are known to weaken hydropatterning (5). Similar to Arabidopsis, we found in maize that auxin accumulates preferentially on the contact-side of hydropatterning roots which creates a bias for lateral root induction (fig. S5). Furthermore, it has been shown that the auxin-response transcription factor AUXIN RESPONSE FACTOR 7 (ARF7) is sumoylated in cells on the air-side of roots, promoting binding to the repressor protein INDOLE-3-ACETIC ACID INDUCIBLE 3 (IAA3) which blocks initiation of lateral root founder cells (18). To identify novel loci and associated genes for hydropatterning with relevance to maize, we conducted Genome and Transcriptome Wide Association Studies (GWAS/TWAS), which associate single nucleotide polymorphisms, or variation in gene expression amongst the study population, to variation in a trait of interest.

**Fig. 3.**
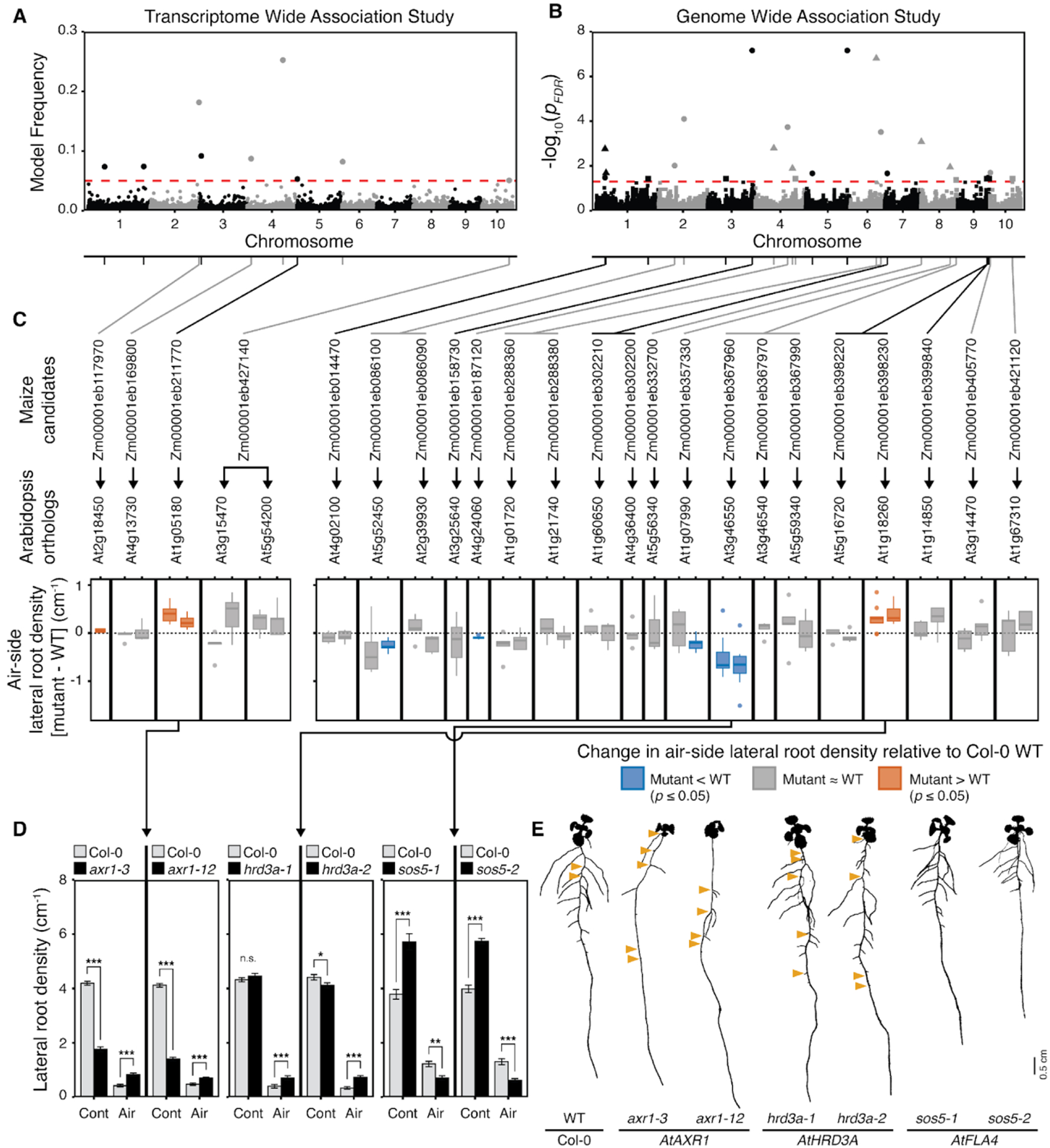
Genetic control for hydropatterning revealed in maize and validated in Arabidopsis. (**A and B**) Manhattan plots of Transcriptome Wide Association Study (TWAS) and Genome Wide Association Studies (GWAS) for air-side lateral root density in maize. (A) TWAS used gene expression data from maize root tips (19). Significance threshold: model frequency = 0.05 (red dashed line). (B) GWAS shows smallest *p*-value for each Single Nucleotide Polymorphism (SNP) across three Minor Allele Frequency cutoffs ≥ 0.4% (solid circles), ≥ 2.2% (solid triangles), and ≥ 4.4% (solid squares). FDR-adjusted significance threshold: *p* = 0.05 (red dashed line). Gray/black lines connect SNPs and associated candidate genes corresponding to the chromosome coloration. (**C**) Validation of maize candidate genes using mutants of corresponding gene orthologs in Arabidopsis. Air-side lateral root densities of mutants are shown relative to Col-0 wild-type (WT). Fill color denotes significant differences (Paired Student’s t-test, *p* ≤ 0.05) mutants > WT (orange), mutants < WT (blue), not significant (gray) *n* = 5 – 10 plates per mutant (5 WT & 5 mutant seedlings/plate). (**D**) Comparisons of contact-side (Cont) and air-side (Air) lateral root densities between Col-0 wild-type (gray) and mutants for *AtAXR1*, *AtHRD3A*, and *AtFLA4* (all mutants black). *n* = 10 plates per mutant (5 WT & 5 mutant seedlings/plate). Asterisks denote significant differences (Paired Student’s t-test: * *p* ≤ 0.05, ** *p* ≤ 0.01, *** *p* ≤ 0.001). Bar graphs: mean +/− SEM. (**E**) Binary images of 11 days-old seedlings. Orange triangles mark air-side lateral roots. Scale bar = 0.5 cm. Dilation was used on images to improve visibility.

TWAS (13) identified nine genes whose expression in germinating seedling roots (19) is significantly associated with differences in air-side lateral root density (Fig. 3A and data S7). Among these TWAS-genes, we found *Zm00001eb211770*, a maize ortholog of *AUXIN RESISTANT 1* (*ZmAXR1*). In Arabidopsis, AXR1 together with E1 C-terminal related 1 (ECR1) act as ubiquitin-activating enzymes that facilitate the RUB modification of CULLIN 1 (CUL1) which is part of the SCF^TIR/AFB^ complex at the center of auxin perception. RUB modification is necessary to allow auxin signal transduction for lateral root induction (20). TWAS in maize found that higher gene expression of *ZmAXR1* is associated with increased air-side lateral root densities (fig. S6A, data S7) which may be due to an increased sensitivity for auxin perception leading to more frequent induction of lateral roots on the air-side. To analyze the origin of the variation in *ZmAXR1* gene expression, we mapped the associated expression Quantitative Trait Loci (eQTL). This analysis revealed several significant expression-associated Single Nucleotide Polymorphisms (e-SNP) (fig. S6B, data S8 and 9). The most significant e-SNP, Chr5_2705946, co-localized with *ZmAXR1* itself, indicating that cis-acting regulatory variation may explain the variation in *ZmAXR1* gene expression associated with air-side lateral root density. Input data for the TWAS suggested expression of *ZmAXR1* in root tips of most inbred lines (fig. S6A). This was confirmed through in situ detection of *ZmAXR1* transcripts in root tips of maize inbred line B73 by Hybridization Chain Reaction (fig. S7, B and C). These results indicate that auxin regulation and related processes play a role in hydropatterning for maize roots as previously demonstrated in Arabidopsis (5, 18).

In parallel, GWAS (21, 22) identified 30 unique Trait-Associated SNPs (TASs) for air-side lateral root density (Fig. 3B, fig. S6 C and D, and data S10), suggesting that variation in hydropatterning is controlled by numerous loci in maize. In almost all cases, higher air-side lateral root density was associated with the less frequent allele (minor allele) of the TAS within our population, except for TAS Chr8_143668219, as shown by the effect estimate (data S10). This supports our hypothesis that weakening of hydropatterning may have been caused by relaxation of selection, since selection typically constrains the occurrence of genetic variants (23). We identified a total of 40 genes within 20-kb windows centered on the TASs. In cases where no gene was located within these windows, the next closest gene was included (data S11). These genes were considered candidates for genes that may control air-side lateral root density in maize.

## Validation of maize candidate genes using Arabidopsis orthologs

We identified maize candidate gene orthologs in Arabidopsis, and screened available mutant lines for hydropatterning defects (fig. S8A and data S12 and 13). Screening revealed seven genes for which at least one mutant allele showed a significant change in air-side lateral root density and percent air-side lateral roots (Fig. 3C and fig. S8B). For three of these genes, two independent mutant alleles both showed significant, matching changes in air-side lateral root density, confirming their association with hydropatterning (Fig. 3, D and E)

Auxin signaling pathway mutants *axr1-3* and *axr1-12* of *AtAXR1* showed significant increases in air-side lateral root density and percent air-side lateral roots compared to the wild-type, while contact-side lateral root density dropped significantly (Fig. 3D and E). This defect is similar to the phenotype of other auxin-pathway mutants in Arabidopsis (5, 18) and confirms that the auxin hormone pathway plays an important role in promoting the bias in lateral root development towards the moisture-contacting side of the root both in Arabidopsis and maize.

Similarly, we observed significant increases in air-side lateral root density and percent air-side lateral roots in Arabidopsis mutants of HMG-CoA REDUCTASE DEGRADATION 3A (*AtHRD3A*), *hrd3a-1* and *hrd3a-2* (Fig. 3D and E). *AtHRD3A*, an ortholog of GWAS candidate *Zm00001eb398230* (*ZmHRD3A*), recruits misfolded proteins for endoplasmic reticulum‐associated protein degradation to the HRD1/HRD3 complex (24). Targets include misfolded receptor-like kinases and glycosylated proteins (25), suggesting that hydropatterning may rely upon proteins acting at the plasma membrane that are also targets of endoplasmic reticulum‐associated protein degradation. In contrast to mutants of *AtAXR1*, *hrd3a-1* and *hrd3a-2* showed no, or a relatively small, changes in contact-side lateral root densities, respectively. This suggests that HRD3A may primarily function in the suppression of air-side lateral root development. In maize, *ZmHRD3A* was discovered as one of three candidate genes associated with TAS Chr9_147576641 (data S11). Mutants of *At5g16720*, an ortholog of *Zm00001eb398220* associated with the same TAS, did not affect hydropatterning (fig. S7B). *ZmHRD3A* is expressed at the root tip in the same region where moisture signals control the patterning of lateral roots (fig. S7, A and D).

In contrast to *axr1* and *hrd3a*, Arabidopsis mutants of *FASCICLIN-LIKE ARABINOGALACTAN-PROTEIN 4* (*AtFLA4*), named *salt overly sensitive5* (*sos5-1* and *sos5-2*), showed significant strengthening of hydropatterning with decreases in air-side lateral root density and percent air-side lateral roots compared to wild-type (Fig. 3D). The mutant *sos5-1* was identified for its defects in growth on saline media (26). While the primary root phenotype under salinity and ionic stress has been studied extensively (27), no reports have described a lateral root phenotype. *AtFLA4* belongs to a group of 21 fasciclin-like arabinogalactan proteins and carries two fasciclin 1 domains that allow it to interact with the extracellular cell wall matrix (28, 29), which may allow it to sense extracellular cues originating from the environment. The decrease in air-side lateral root density in the *sos5* mutants was accompanied by a significant increase in contact-side lateral root density and a significant reduction in primary root length (Fig. 3E and fig. S9A). Taking this reduction of primary root length into account, *sos5-1* and *sos5-2* both showed a significant decrease in the total number of emerged air-side lateral roots per seedling but only a small or no increase in total contact-side lateral roots per seedling (fig. S9B). Thus, these data indicate that *sos5* primarily suppresses air-side lateral roots, while changes in contact-side lateral root density may result from pleiotropic effects on root length. *AtFLA4* is an ortholog of GWAS candidate *Zm00001eb367960* (*ZmFLA4*), which was discovered as one of four candidate genes associated with TAS Chr8_175425640 (data S11). Similar to *AtFLA4*, *ZmFLA4* contains two fasciclin 1 domains (30). Orthologous Arabidopsis mutants of two other candidate genes associated with the same TAS, *Zm00001eb367970* and *Zm00001eb367990*, showed no defect in hydropatterning (fig. S8B). This provides evidence that variation associated with *ZmFLA4* may be the primary determinant of the observed variation in hydropatterning at TAS Chr8_175425640. In maize, *ZmFLA4* is expressed at the root tip (fig. S7, A and E).

## Ethylene as an air-side signal mediating hydropatterning

**Fig. 4.**
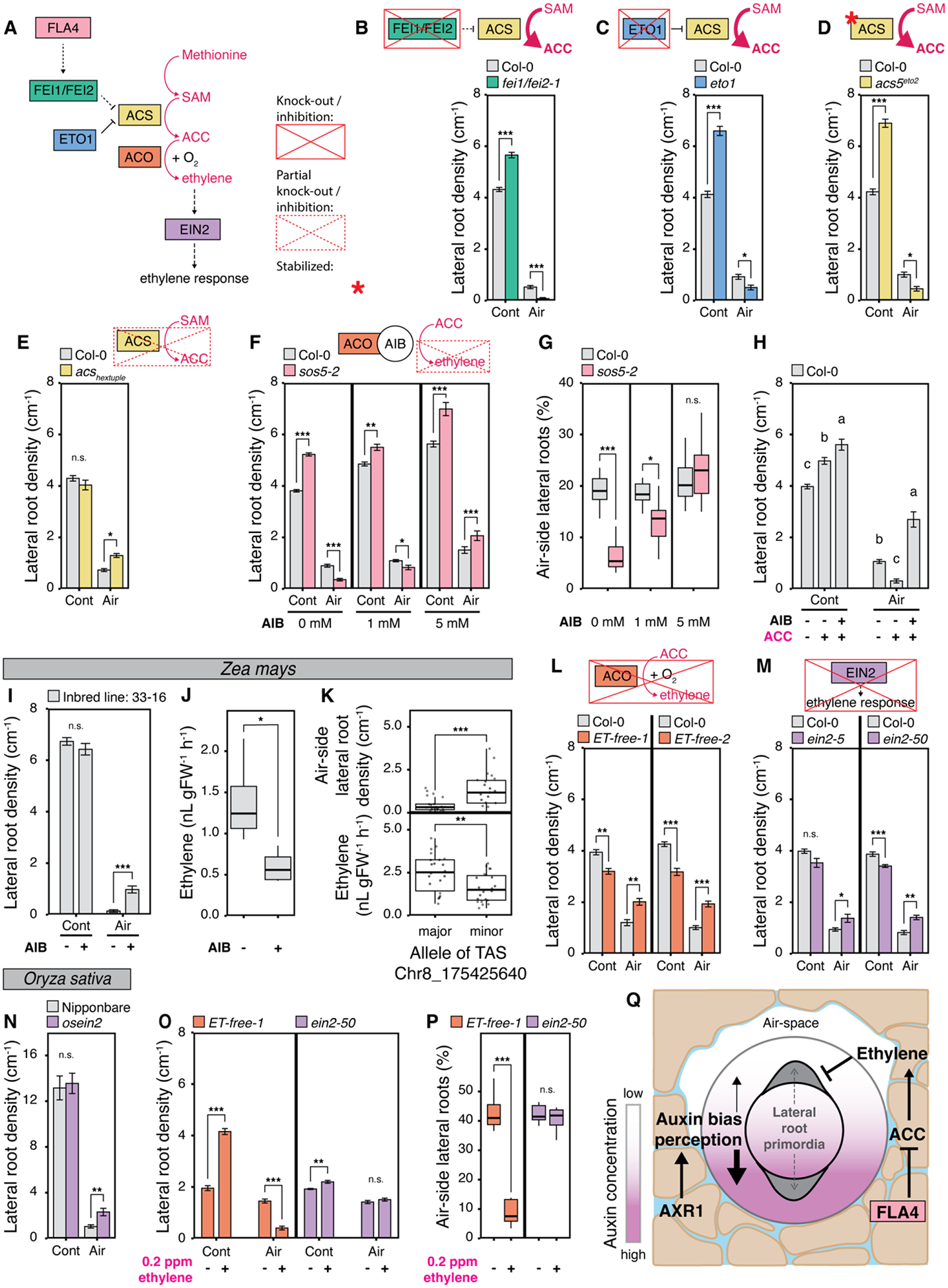
Ethylene inhibits formation of air-side lateral roots in Arabidopsis, maize, and rice. (**A**) FLA4 and the ethylene pathway in Arabidopsis (27). (**B - E**) Contact- (Cont) and air-side (Air) lateral root densities in Arabidopsis mutants (C) *eto1* (blue, n = 5 plates), (D) *acs5*^*eto2*^ (yellow, n = 5 plates), and (E) *acs2-1/4-1/5-2/6-1/7-1/9-1* hextuple mutant (yellow, n = 5 plates) relative to Col-0 (gray). (**F and G**) Effect of 2-Aminoisobutyric acid (AIB) on Arabidopsis mutant *sos5-2* relative to Col-0 (pink, n = 10 plates/treatment). (**H**) Arabidopsis Col-0 response to ACC, AIB, and combination: (–) mock control and (+) 0.05 mM ACC / 5 mM AIB; n = 10 plates/treatment. Letters denote significant differences between treatments (ANOVA, post-hoc Tukey HSD Test). (**I and J**) Response of maize inbred 33–16 to (+) 10 mM AIB relative to (–) mock control (n = 20 plants/treatment), and related ethylene production (n = 4 samples of five roots/treatment). (**K**) Air-side lateral root density and root ethylene production for maize inbred lines (n = 22/allele) with the major (G) or minor (T) allele at TAS Chr8_175425640. (**L - N**) Contact- and air-side lateral root densities in Arabidopsis mutants (L) *ET-free-1* & *ET-free-2* (orange, n = 8 – 9 plates) and (M) *ein2-5* & *ein2-50* (purple, n = 10 plates) relative to Col-0, and in (N) rice mutant *osein2* (purple, n = 11 seedlings) relative to Nipponbare wild-type (gray, n = 16 seedlings). (**O and P**) Effect of exogenous ethylene treatment on Arabidopsis mutants *ET-free-1* (n = 8 plates/treatment) and *ein2-50* (n = 8 plates/treatment). (**Q**) Working model of hydropatterning controlled by auxin and ethylene. Asterisks denote significant differences (Student’s t-test: * *p* ≤ 0.05, ** *p* ≤ 0.01, *** *p* ≤ 0.001), n.s., not significant. Bar graphs: mean +/− SEM.

Ethylene is a gaseous plant hormone that regulates development in response to several abiotic stresses (31). Accumulation of root-produced ethylene in compacted soils serves as a signal leading to root growth inhibition (32). In Arabidopsis, *AtFLA4* may act in a genetic pathway regulating the synthesis of ethylene precursor 1-aminocyclopropane-1-carboxylate (ACC) from S-adenosylmethionine (SAM) (Fig. 4A) (27). In this pathway, AtFLA4 acts upstream of two leucine-rich repeat receptor-like kinases, AtFEI1 and AtFEI2 (33). These kinases interact with 1-AMINOCYCLOPROPANE-1-CARBOXYLATE SYNTHASE (ACS) 5 and 9, which are involved in the synthesis of ACC. Associated with this pathway, ETHYLENE OVERPRODUCER 1 (AtETO1) functions as a negative regulator of type-2 ACS enzymes, including ACS5 and ACS9 (34).

We found that double mutants of *AtFEI1* and *AtFEI2* (*fei1*/*fei2-1*) as well as single mutants of *AtETO1* (*eto1*) and *AtACS5* (*acs5*^*eto2*^, which carries a C-terminal mutation in ACS5 that increases protein stability) all showed similar reductions in air-side lateral root density as observed in the *AtFLA4* mutants *sos5-1* and *sos5-2*. Concomitantly, all mutants showed an increase in contact-side lateral root density and a reduction in primary root length (Fig. 4, B to D, and fig. S9, C to E). Screening of the single mutants *fei1* and *fei2-1* revealed no significant effects on air-side lateral root density (fig. S9F), corroborating a suggestion that both genes may act in a redundant fashion (33). While both *eto1* and *acs5*^*eto2*^ increase ACC synthesis, a reduction of ACC synthesis in the *AtACS* hextuple mutant (*acs2-1, acs4-1, acs5-2, acs6-1, acs7-1, acs9-1*) led to a significant increase in air-side lateral root density (Fig. 4E). We also tested the single mutant *acs5-1*, but observed no difference compared to wild-type (fig. S9G). This result is likely due to the high-level of redundancy between *ACS* genes in Arabidopsis, which has eight functional ACS homologs (35). Our results suggest that genetic modulation of ACC synthesis affects hydropatterning in a way that resembles the phenotypes of *sos5-1* and *sos5-2*.

Next, we asked whether ACC itself or ethylene, which is synthesized from ACC by ACC-oxidases (ACOs), causes the observed repression in air-side lateral root development. We found that treatment with 2-aminoisobutyric acid (AIB), a competitive inhibitor of ACOs, caused an increase in air-side lateral root density and rescued the *sos5-2* mutant phenotype (Fig. 4, F and G). Likewise, treatment of Col-0 wild-type with ACC alone reduced air-side lateral root density, likely due to the increased production of ethylene since this effect was reversed by co-treatment with AIB (Fig. 4H). Both ACC and ACC + AIB treatments showed significant increases in contact-side lateral root density compared to mock treated Col-0 wild-type. These increases could be due to a concomitant reduction in primary root length with both treatments (fig. S9H), and/or potential effects of ACC itself on lateral root induction (36). Taken together, these observations suggest a central role for ethylene in the suppression of air-side lateral root development, which does not require localized ACC synthesis, since exogenous ACC application on the contact-side was able to induce the same effect.

In maize, AIB treatment of a strong hydropatterning inbred line 33–16 increased air-side lateral root density and significantly reduced ethylene production (Fig. 4, I and J). This indicates that ethylene suppresses air-side lateral root development in maize as well. Measurements of ethylene production from root tips of maize inbred lines that either carry the major or minor allele for TAS Chr8_175425640, localized about 2 kilobases upstream of *ZmFLA4*, showed the minor allele was associated with lower ethylene production and higher air-side lateral root densities (Fig. 4K). This may suggest that TAS Chr8_175425640 is linked to genetic variation that affects *ZmFLA4* function leading to the observed differences in ethylene production.

Complete disruption of ethylene synthesis in two *AtACO* quintuple mutants (*ET-free-1* and *ET-free-2*), in which all five *AtACO* genes were mutated by CRISPR/Cas9 (37), showed a significant increase in air-side lateral root density (Fig. 4L). Similarly, ethylene perception mutants *ein2-5* and *ein2-50* showed a significant increase in air-side lateral root density (Fig. 4M). Together these observations confirm that ethylene suppresses air-side lateral root development. Concomitantly, we observed a decrease in contact-side lateral root density for *ET-free-1*, *ET-free-2*, *ein2-5*, and *ein2-50* that mirrored the increase in air-side lateral root density leading to no change in total lateral root density (fig. S9, I and J). Since lateral root induction can only occur at one of the two xylem poles in Arabidopsis (38), it is possible that a derepression of air-side lateral root development in these mutants leads to lateral root redistribution from the contact-side. While mutants for *EIN2* have not been described in maize, a mutant allele of *OsEIN2* in rice was available and showed a similar defect in hydropatterning as the Arabidopsis mutant alleles (Fig. 4N), providing further evidence that ethylene-dependent regulation of hydropatterning is conserved between Arabidopsis and grasses. A double mutant of ETHYLENE-INSENSITIVE3 (EIN3) and ETHYLENE-INSENSITIVE3-LIKE 1 (EIL1), *ein3/eil1*, two major transcriptional regulators of ethylene signaling that act downstream of EIN2, showed no difference in air-side lateral root density compared to Col-0 wild-type (fig. S9K). This might be due to redundancy in transcriptional regulation or an alternative pathway (39) that leads to air-side suppression of lateral root development by ethylene. Exogenous treatment of *ET-free-1* and *ein2-50* with 0.2 ppm ethylene was sufficient to suppress air-side lateral root development in *ET-free-1*, while *ein2-50* showed no response (Fig. 4, O and P). Our results suggest that localized ethylene synthesis is not necessary and that additional mechanisms must exist that control air-side specific suppression of lateral roots by ethylene.

Since auxin is necessary for lateral root induction and ethylene has been shown to induce local auxin biosynthesis (40), we generated a double mutant of *sos5-2* and *axr1-3* to study their epistatic interactions. While the disruption of auxin signaling leads to an increase in air-side lateral roots, the double mutant *sos5-2*/*axr1-3* showed a significant decrease in air-side lateral root density (fig. S9L). Our results indicate an additive interaction between auxin and ethylene responses. Further work will be necessary to elucidate how air-side lateral root suppression by ethylene is connected to auxin controlled lateral root induction on the contact-side.

## Conclusions

Our results reveal that hydropatterning is a crop-relevant response of roots to heterogeneity in soil moisture. Development of modern breeding germplasm in maize led to the weakening of hydropatterning in temperate regions, likely through relaxation of selection. This divergence in hydropatterning may relate to the different selection pressures experienced by each subpopulation. Through genetic analyses, we detected associations between hydropatterning and the auxin and ethylene signaling pathways. Dissection of these pathways in maize, rice and Arabidopsis demonstrates that auxin signaling promotes a bias in lateral root development towards moisture-contacting surfaces of the root, while ethylene suppresses branching on air-exposed surfaces (Fig. 4Q and fig. S9M). FLA4, acting at the top of a signaling pathway that restricts ethylene biosynthesis, may perceive an as-of-yet unknown environmental cue to tune the degree to which root architecture is responsive to local soil structure and water availability. Further genetic work in maize is needed to validate our finding from Arabidopsis. A deeper understanding of these pathways may allow for the control of moisture-responsive root growth to improve drought resilience in maize.

## Supplementary Material

Data tables **data S1.** *Zea mays* inbred lines obtained from the USDA-ARS National Plant Germplasm System (accession group: Maize.Set.Inbred.Diversity.282.Plus.NAM.Parents).
**data S2.** Phenotypes recorded from primary roots in the hydropattering assay. Data are shown as median and standard deviation (SD).
**data S3.** Phenotypes recorded from primary and crown roots in the hydropattering assay. Data are shown as median and standard deviation (SD).
**data S4.** Correlation analysis between field-grown maize traits (10.1093/gigascience/giac080) and hydropatterning traits taking population structure as a covariate into account.
**data S5.** Ancestry components of *n* = 231 inbred lines calculated by ADMIXTURE (k=3). Sweet corn (sweet) and popcorn were defined a priori and not included in the analysis.
**data S6.** Statistical summary of global and subpopulation pair-wise *Q*_ST_ - *F*_ST_ comparisons.
**data S7.** Summary statistic of Transcriptome Wide Association Study (TWAS) for air-side lateral root density at model frequency > 0.05.
**data S8.** Summary statistics of expression Quantitative Trait Loci (eQTL) for *AUXIN RESISTANT 1* (*ZmAXR1*; *Zm00001eb211770*) at FDR < 0.05.
**data S9.** Expression Associated SNPs (e-SNP) for *AUXIN RESISTANT 1* (*ZmAXR1*; *Zm00001eb211770*) and co-located SNP-genes within a 20 kb window.
**data S10.** Combined summary statistics of Genome Wide Association Study (GWAS) for air-side lateral root density at FDR < 0.05 for three MAF cutoffs (≥ 0.004, ≥ 0.022, ≥ 0.044).
**data S11.** Maize Trait Associated SNPs (TAS) for air-side lateral root density and co-located SNP-genes within a 20 kb window.
**data S12.** Arabidopsis mutant alleles used for orthologous validation of maize candidate genes from TWAS and GWAS.
**data S13.** Statistical summary of mutant analysis in Arabidopsis.
**data S14.** Arabidopsis mutant alleles used for the investigation of the ethylene pathway.
**data S15.** Best Linear Unbiased Predictions (BLUPs) of hydropatterning traits.
**data S16.** Hybridization Chain Reaction probes for maize inbred B73. Individual probes for each target were combined.

1

## Data Availability

Data and summary statistics are available in the supplementary materials. Software, raw data and code for image processing, to generate data, statistics, and figures are available on Dryad and Zenodo (41, 42).
