# Supplementary material for: Moisture-responsive root-branching pathways identified in diverse maize breeding germplasm
Source: Science. Author manuscript; Available in PMC 2025 Mar 31. (PMC11956805; doi:10.1126/science.ads5999)
Supplement: 1 [file NIHMS2065295-supplement-1.pdf]

Supplementary materials

Materials and Methods

Plant materials

Zea mays (maize) inbred lines were obtained from the USDA-ARS National Plant Germplasm System (accession group: Maize.Set.Inbred.Diversity.282.Plus.NAM.Parents) (data S1). Selected seed stocks were bulked up by sib-pollination of plants that were grown in a field at Stanford, CA.

Arabidopsis thaliana (Arabidopsis) mutants for orthologs of maize candidate genes were obtained from the Arabidopsis Biological Resource Center (ABRC) (data S12). Additional Arabidopsis mutants that were needed to examine the effect of ethylene on hydropatterning were either obtained from the ABRC or from collaborators and certain crosses were made in house (data S14). For bulking up seeds, selection of homozygous plants, and crosses, Arabidopsis seeds were surface sterilized for 10 min. using a 25% (v/v) bleach (Clorox, Product No. 4460032263) and 0.1% Tween® 20 solution (Sigma-Aldrich, CAS No. 9005-64-5) and then washed four times with sterile water. Sterile seeds were then planted on solid media plates with 4.3g/L Murashige & Skoog Salts (Caisson Labs, Ref. No. MSP01-50LT), 1% sucrose (Sigma-Aldrich, CAS No. 57-50-1), 0.05% MES Hydrate (Sigma-Aldrich, CAS No. 1266615-59-1), and 0.4% Gelzan (Sigma-Aldrich, CAS No. 71010-52-1), and stratified for 72 h at 4 ℃ in the dark. Plates were transferred to a growth chamber and grown under long-day conditions (16 hours light / 8 hours dark, 22 ℃, 37% relative humidity) for 10 days after which they were either transferred to soil directly or genotyped for homozygous T-DNA insertions before transfer to soil. Seedlings were grown on PRO-MIX HP soil (Hummert International, Item No. 10200400) to maturity in a growth chamber under long day conditions (16 hours light/8 hours dark, 22 ℃, 50% relative humidity). Seeds were collected and stored at room temperature until further use in experiments. Mutant alleles and crosses were confirmed via Polymerase Chain Reaction (PCR) and subsequent analysis via gel electrophoresis. Primers were designed using either the SIGnAL tool (*43*) or Geneious Prime 2023.2.1 (Dotmatics, New Zealand) (data S12). PCR was performed using the Phire Plant Direct PCR Master Mix Kit (Thermo Fisher, Cat. No. F160S). For mutants with point mutations, subsequent Sanger sequencing was carried out to confirm the genotype of the plants.

Maize hydropatterning assay

Design: The phenotyping system was adapted from *GrowScreen-PaGe* (*44*). With the aim to achieve a uniform moisture gradient across the primary root and hydropatterning of lateral root branches, we designed a custom-built hydropatterning assay which uses acrylic panels for structural support (fig. S1). In contrast to gellan gum based hydropatterning assays in plates that have been previously described (*9*), our germination-paper based assay is less prone to fungal contamination, since no nutrients are added, and roots have more space to grow. The hydropatterning assay consists of three components (fig. S1A): (1) Rhizo-sheets that were made from laser cut 1/8” black acrylic sheets (Calsak Plastics, Item No. 61299) as a structural support (Sheet.svg). Acrylic sheets were covered on both sides with one layer of pre-moistened Ahlstrom Grade 222 Blot Paper (Great Lakes Filters, SKU: 2228) and one layer of 76# Heavy-Weight Seed Germination Paper (Anchor Paper Company, Item No. SD7630) on the outside. The germination paper was folded twice to form a channel to hold the germinating seeds (fig. S1B).

Two small triangular cut-outs were introduced 2.25” from each side at the bottom of the channel to allow the roots to pass through. Sheets were inserted into a pouch made from Phifer BetterVue window screen (Phifer, Item No. 3024751) to prevent roots from growing away from the surface. Each rhizo-sheet was designed to hold up to four seedlings. (2) Rhizo-racks were made from laser cut 1/4” black acrylic sheets (Calsak Plastics, Item No. 61308). Each rack was assembled from three pre-cut sheets (Rack.svg) that were connected using four 3/8"-16 PVC Threaded Rods (McMaster-Carr, Item No. 98871A370) and a total of 24 3/8"-16 PVC Hex Nuts (McMaster-Carr, Item No. 94806A031). Each rack was designed to hold six rhizo-sheets (3) Black polyethylene 12 gallon tanks (Plastic-Mart, Item No. R121812A) were used to hold the rhizo-racks with rhizo-sheets and served as a reservoir for water to keep the filter and germination papers moist (fig. S1C). Each tank was covered with a laser cut 1/8” clear acrylic sheet (Calsak Plastics, Item No. 61413) to reduce water loss by evaporation.

Phenotyping: Maize seeds were rehydrated for 4 hours in tap water at room temperature. Once rehydrated, they were incubated for 5 min. in a water bath at 56 ˚C before being surface sterilized for 10 min. using a 20% (v/v) bleach (Clorox, Product No. 4460032263) and 0.1 µL mL^{-1} Tween® 20 solution (Sigma-Aldrich, CAS No. 9005-64-5). Afterwards they were immediately washed 5-7 times with sterile water.
Primary root assay setup: Sterilized maize seeds were germinated on 76# Heavy-Weight Seed germination paper (Anchor Paper Company, Item No. SD7630) in 120 x 120 x 17 mm square vented polystyrene petri dishes (Greiner Bio-One, Item No. 688102) for 2 days in a black polyethylene box (Plastic-Mart, Item No. R121812A) in the dark in a walk-in growth chamber (*45*) (fig. S1D). Conditions in the growth chamber were set to 30 ˚C day / 22 ˚C night (16-hour photoperiod, 250 µmol m^{-2} s^{-1} light intensity, 50% relative humidity). In total, we were able to successfully germinate seeds of 285 inbred lines from a total set of 293 inbred lines (data. S1). Once primary roots started to emerge (5 to 10 mm in length), seedlings were transferred into the hydropatterning assay described above and grown for 10 days in the same growth chamber. Positions were randomly assigned with each inbred line being present at least once in each batch.
Crown root assay setup: Sterilized seeds were germinated in paper rolls made from 76# Heavy-Weight Seed germination paper (Anchor Paper Company, Item No. SD7630) and grown in a growth chamber set to 30 ˚C day / 22 ˚C night (16-hour photoperiod, 250 µmol m^{-2} s^{-1} light intensity, 50% relative humidity) until crown roots emerged. Once crown roots emerged and had grown 1 to 2 cm in length, seedlings were transferred into the hydropatterning assay described above and grown for 10 days in the same growth chamber.
Imaging: After 10 days, when lateral roots along the entire length of the primary/crown root on the rhizo-sheets had emerged, 12 cm segments of primary/crown roots were harvested and digitized (contact- and air-side) using a Epson Perfection V800 Photo Scanner (Seiko Epson Corporation, Model B11B223201) with the following settings: Photo Mode, Reflective, 24-bit Color, 1200 dpi, Image Format: TIFF.

Image analysis: Images were prepared for counting of lateral root emergence points using a series of custom macros in ImageJ/Fiji (*41*, *46*). First digitized roots were separated into individual files (*00_ImageSeparation.ijm*) and corresponding air- and contact-side root segments were aligned (*01_RegisteredStacks.ijm*, *01_Register.py*). Then, root segments were traced, measured, and straightened (*02_SegmentTrace.ijm*). Lateral root emergence points along the contact- and air-side of primary/crown root segments were counted manually using the *Multi-point* tool in ImageJ/Fiji. Counts and coordinates of lateral roots were exported using the *Analyze* > *Measure* commands.

Data analysis and visualization: Lateral root counts from images were imported into R version 4.2.1 (*47*). Data analysis and visualization were performed with the help of the *tidyverse* R package (*48*). Contact- and air-side lateral root densities were calculated as the sum of all contact- or air-side lateral roots divided by the length of the root segment that they were counted on using the script *MaizeHydropatterning_PhenoAnalysis.Rmd* (*41*). Percent air-side lateral roots was calculated as the sum of all air-side lateral roots divided by the total sum of all lateral roots and multiplied by 100. Median and standard deviation were calculated for biological replicate measurements and used for visualization (data S2). After removing roots of inbred lines that showed abnormal growth or had less than 20 lateral roots on their root segments, we retained phenotypes for 250 inbred lines. Best Linear Unbiased Prediction (BLUP) values for contact- and air-side lateral root density as well as percent air-side lateral roots were calculated by fitting linear mixed models using the lme4 R package (*49*) and treating the box of seedlings as a fixed effect and genotype as a random effect (data S15).

<u>Microscopy of lateral root primordia</u>

Maize seedlings were grown in the hydropatterning assay for 3 - 4 days before collecting 7 cm sections of primary roots for microscopy. To be able to distinguish the contact- and air-side after collection, 1 cm^2 pieces of window mesh were glued with PELCO® Pro CA44 Tissue Adhesive (TED PELLA INC., Prod. No. 10033) to the air-side of the root sections at their distal end.

Lateral root primordia quantification: Root segments were fixed in 4% paraformaldehyde (Electron Microscopy Sciences, SKU: 15710) for an hour under vacuum, rinsed four times in 1X phosphate buffered saline (PBS) (VWR International, Item No. MRGF-6235), and transferred to ClearSee [10% Xylitol (Sigma, CAS-No: 87-99-0), 15% Sodium deoxycholate (Sigma, CAS-no: 302-95-4), 25% Urea (Sigma, CAS-No: 57-13-6)] (*50*) with gentle agitation for two weeks. Cleared samples were stained with SYBR GREEN (Sigma-Aldrich, Item No. B0904) (1:1000 1X PBS) to mark nuclei and Basic Fuchsin (Sigma-Aldrich, Item No. S9430) as a counterstain (*50*). SYBR GREEN staining allows the visual identification of areas of rapidly dividing cells such as lateral root primordia due to the high density of stained nuclei in these areas (*51*). Samples were mounted on slides with proper orientation considering the glued mesh piece and imaged on a stereo microscope Leica M205 FCA equipped with 1X PlanApo objective and a fluorescence system with ETGFP and ETDSR filter sets (Leica Microsystems THUNDER Imager Model Organism). Individual frames were stitched together using the Leica LasX Navigator software.

Longitudinal sections: To obtain longitudinal sections for detailed images of lateral root primordia (Fig. 1A), primary root sections were glued to a Vibratome stage using PELCO® Pro CA44 Tissue Adhesive (TED PELLA INC., Prod. No. 10033) and embedded in 8% UltraPure™ LMP Agarose (Invitrogen, Cat. No. 16520-100). 100 µm thick, longitudinal root tissue sections were cut from root tips using a Vibratome Series 1500 (The Vibratome Company, discontinued). Immediately after, sections were transferred to ClearSee [10% Xylitol (Sigma, CAS-No: 87-99-0), 15% Sodium deoxycholate (Sigma, CAS-no: 302-95-4), 25% Urea (Sigma, CAS-No: 57-13-6)] (*50*) for 24 h, then stained with Calcofluor White (Sigma-Aldrich, Item No. 18909) for 60 min. and SYBR GREEN (Sigma-Aldrich, Item No. B0904) for 30 min. Finally, sections were mounted on slides and imaged using a Leica SP8 confocal microscope equipped with a Glycerin-immersion 20X objective. Calcofluor White was excited at 405 nm and detected at 425-475 nm while SYBR GREEN was excited at 488 nm and detected at 510-550 nm.

Correlation analysis

Partial correlation analysis was performed to correlate BLUPs (data S15) of hydropatterning traits (contact-side lateral root density, air-side lateral root density, and percent air-side lateral roots) with BLUPs of phenotypic traits from field-grown maize plants collected in a meta-analysis (*11*). In R, partial Pearson's correlation coefficients were calculated using the *ppcor* library (*52*) in the script *MaizeHydropatterning_PartialCor.Rmd* (*41*). Principal components PC1 and PC2 of the population structure (see below) were included as covariates. FDR-adjusted *p*-values were calculated, and the significance threshold was set to $p \leq 0.05$.

X-ray Computed Tomography imaging

Non-destructive assessment of lateral root formation in soil was made using a modified version of the method of (*5*). A Newport series loamy sand (sand 83.2%, silt 4.7%, and clay 12.1%; pH 6.35; organic matter 2.93%; FAO Brown Soil) collected from the University of Nottingham farm at Bunny, Nottinghamshire, UK (52.8586°, -1.1280°), air dried and sieved to <2 mm, was packed into PVC columns (52 mm diameter x 150 mm length) to a typical field bulk density of $1.3\ g^{-1}\ cm^{3}$. Ten replicate columns were saturated with water from the bottom and then allowed to freely drain for two days to notional field capacity. To compare lateral root formation between soil cores with or without a macropore (to give a scenario where the root has only partial contact with a moist soil surface), a macropore was created from the center of each core using a 7 mm diameter cork borer for half of the replicates. A pre-germinated maize kernel was placed on a 15 mm disk of nylon mesh at the either the center (no macropore samples) or above the macropore and then covered with moist soil taking care not to fill the macropore with soil. The nylon mesh stopped loose soil falling down the macro pore. Plants were subsequently grown for 5 days at 25 °C day / 21 ˚C night (16-hour photoperiod, 250 µmol $m^{-2}\ s^{-1}$ light intensity, 50% relative humidity) in a growth cabinet (Conviron, A1000) before X-ray CT scanning. The water content of the soil at field capacity is moist (approximately 26%), therefore, it is expected that the air within the macropore would have a humid microclimate. A v|tome|x m 240 kV (Waygate Technologies, Germany) X-ray CT system was used to scan each soil core. Scan settings were 160kV X-ray potential energy, 200 µA current, 1 mm aluminum filter on the X-ray tube, 200 ms detector timing, 3000 projection images in FAST scan mode (continuous rotation, no image averaging). Scan resolution was 60 microns with each scan taking 10 min. to acquire. Projection images were reconstructed to 3D volumes using DatosREC software (Waygate Technologies, Germany). Lateral root positions were quantified by manual assessment of the 3D volumes in VGStudioMAX software (Volume Graphics GmbH, Germany). Six strong and weak hydropatterning maize inbred lines were screened (strong: B73, CI64, 33-16; weak: T8, MS153, OH7B).

Population structure analysis and Q_{ST} - F_{ST} comparisons

Genomic variant selection: Variants for all phenotyped maize inbred lines and selected outgroups were extracted from the *Zea mays* HapMap v3.2.1 data available on the CyVerse Data Commons (*53*). In total 231 maize inbred lines were extracted from the "282_onHmp321" and "hmp321_unimputed" VCF files, respectively, with the vcftools (v0.1.16) '--keep' option (*54*), and merged with bcftools (v1.15.1) 'merge' (*55*). We then filtered sites with vcftools to retain only biallelic Single Nucleotide Polymorphisms (SNPs) (--remove-indels --min-alleles 2 --max-alleles 2), remove sites with more than 10% missing data and a minor allele frequency below 0.01 (--max-missing 0.9 --maf 0.01), and prune variants within a 12kb distance from each other (--thin 12000). Since we were interested in keeping only neutrally evolving SNPs to infer

population structure and the neutral F_{ST} distribution, we removed any SNPs falling within the pruning distance from coding regions using bedtools (v2.30.0) ‘window’ (*56*). A distance thinning method was chosen, as pruning based on correlations between SNPs can bias F_{ST} estimates in structured populations without a homogenous LD distribution, and lead to a more liberal Q_{ST} - F_{ST} estimation (*57*). The pruning distance was chosen based on estimates of Linkage Disequilibrium (LD) decay in tropical maize, which drops to $r^2 = 0.2$ at a distance of ~ 4kb and $r^2 = 0.1$ at a distance of ~ 11.6kb (*58*). In total, 74,022 neutral non-coding SNPs were retained for further analysis.

Population structure analysis: We examined the population structure in the data set using principal components analysis in PLINK v1.9 (*59*). Genetic clustering was assessed with ADMIXTURE v1.3.0 (*60*) and the results were compared to those of previous studies using microsatellite markers (*8*, *61*). We decided on the final number of clusters by considering the lowest cross-validation error but also considering what is known about maize subpopulations from the literature. Tropical/subtropical (ts), stiff stalk (ss), and non-stiff stalk (nss) subpopulations were defined by a 0.8 cutoff for the majority ancestry components, while popcorn and sweetcorn were defined a priori due to small sample sizes as previously reported (*8*). Inbred lines without major ancestry components (> 0.8), were defined as mixed.

Q_{ST} - F_{ST} *comparisons*: We calculated global and pairwise F_{ST} for the same variants used for population structure analysis, excluding those with a Minor Allele Frequency (MAF) below 0.1. We imposed this additional filter as variants with a low MAF are known to decrease estimates of F_{ST} and choosing a higher MAF threshold would therefore make our Q_{ST}-F_{ST} tests more conservative. F_{ST} was estimated according to the definition of Weir & Cockerham (*62*).
We converted the VCF file with the vcftools ‘--012’ option to create a genotype matrix and substituted ‘-1’ values for ‘9’ to indicate any missing data. This genotype matrix was used to calculate global F_{ST} with the ‘MakeDiploidFSTMat’ function of the OutFLANK package (*63*) in R. Pairwise F_{ST} was calculated with the vcftools (v0.1.16) ‘--weir-fst-pop’ option. Both global and pairwise F_{ST} values were weighted prior to comparison with Q_{ST} values.
Global and pairwise Q_{ST} estimates were calculated for the BLUPs (data S15) of each trait (air-side lateral root density, contact-side lateral root density, and percent air-side lateral roots) using the ‘Pst’ function of the R package Pstat (*64*). A 95% confidence interval was constructed around these estimates by performing 1000 bootstrap replicates using the ‘BootPst’ function of the same package. Distances between the F_{ST} and Q_{ST} estimates were calculated both globally (once for all populations including mixed individuals and once excluding mixed individuals) and for each population pair. Differences were considered potentially significant when the confidence interval of Q_{ST} did not overlap with the weighted F_{ST}. To assign levels of significance, we calculated which percentage of the Q_{ST} and F_{ST} distributions overlapped, with increasing levels from <5% to <1% and <0.1% of overlap, and corrected the values for multiple testing using the Benjamini & Hochberg correction for all pairwise comparisons (*65*). All calculations were done using the script *Qst-Fst_06-12.html* (*41*).

Genome and Transcriptome Wide Association Studies

GWAS: Genome Wide Association Studies (GWAS) were conducted using the FarmCPUpp (*22*), a C++ implantation of the FarmCPU model (*21*). Best Linear Unbiased Prediction (BLUP) values for air-side lateral root density were calculated by fitting linear mixed models using the lme4 R package (*49*) by treating the box of seedlings as fixed effect and genotype as random

effect. A total of 1.2 millions of high-density SNPs that were available for 227 of the 250 phenotypes maize inbred lines (*66*) were used as markers for conducting GWAS using the script *MaizeHydropatterning_FarmCPUpp.GWAS.R* (*41*). Three GWAS runs were conducted using MAF cutoffs ≥ one inbred line (0.4%), ≥ five 5 inbred lines (2.2%), or ≥ 10 inbred lines (4.4%) with the minor allele to identify a spectrum of common to rare trait-associated genetic variants (fig. S6C). The first three principal components were calculated using the TASSEL 5.0 software (*67*) and were included as covariates to control for population structure. Trait Associated SNPs (TAS) were selected using false discovery rate (FDR) < 0.05 in R version 4.2.1 (*47*). Linkage disequilibrium between TAS was calculated using PLINK v1.9 (*59*). Multiple TAS that were found in close proximity to each other on chromosomes 3 and 10 showed strong associations between alleles (r^2 ≥ 0.8, fig. S6D), respectively. Hence, these are likely associated with the same causal variants. Data analysis and visualization were performed with the help of the *tidyverse* R package (*48*) using the script *MaizeHydropatterning_gwasAnalysis.Rmd* (*41*). For Manhattan plots, the top 40,000 SNPs with the smallest *p*-values were subsampled to create images of a manageable size. Likewise, for quantile-quantile plots, data was subsampled. SNP-genes within 20-kb windows centered on the TAS were identified using B73 version 5 reference gene models (*68*).

TWAS: In addition to GWAS, eRD-GWAS (*13*), a Bayesian-based version of Transcriptome Wide Association Study (TWAS) was conducted using the script *MaizeHydropatterning_TWASrun41000.in*p (*41*) and RNA-Seq data from root tips of matching 207 maize inbred lines (*19*). Using an arbitrary Model Frequency threshold ≥ 0.05, significant TWAS-genes were selected in R version 4.2.1 (*47*). Data analysis and visualization were performed with the help of the *tidyverse* R package (*48*) using the script *MaizeHydropatterning_twasAnalysis.Rmd* (*41*).

<u>Expression Quantitative Trait Loci</u>

Expression Quantitative Trait Loci (eQTL) mapping for the expression of *Zm00001eb211770* was performed using the FarmCPUpp (*22*), a C++ implantation of the FarmCPU model (*21*). A total of 1.2 millions of high-density SNPs (*66*) of 207 maize inbred lines with matching gene expression data were used as markers for conducting eQTL mapping. The first three principal components were calculated using the TASSEL 5.0 software (*67*) and were included as covariates to control for population structure. Expression associated SNPs (e-SNPs) were selected using false discovery rate (FDR) < 0.05. Expression associated SNPs (e-SNPs) were selected using false discovery rate (FDR) < 0.05 in R version 4.2.1 (*47*). Data analysis and visualization were performed with the help of the *tidyverse* R package (*48*) using the script *MaizeHydropatterning_eqtlAnalysis.Rmd* (*41*). For Manhattan plots, the top 40,000 SNPs with the smallest *p*-values were subsampled to create images of a manageable size. Likewise, for quantile-quantile plots, data was subsampled. SNP-genes within 20-kb windows centered on the TAS were identified using B73 version 5 reference gene models (*68*).

<u>Identification of maize gene orthologs in Arabidopsis</u>

Maize gene orthologs in Arabidopsis were identified using Phytozome annotations in MaizeGDB (*68*) or directly through Phytozome 13 (*69*). In some cases protein sequence orthologs were narrowed down using BLAST (*70*).

Hybridization Chain Reaction

Hybridization Chain Reaction probes (data S16) were purchased (Molecular Instruments, Inc., USA). Seeds of maize inbred line "B73" were surface sterilized and germinated before transfer to the hydropatterning assay as described above. Once roots had grown in the assay for three days, 2 cm sections of primary root tips were harvested for sectioning directly into FAA fixative (4% formaldehyde, 5% glacial acetic acid, 50% ethanol) on ice. Immediately after, root samples were fixed under vacuum for 1 hour on ice. After fixation, root tips were glued to a Vibratome stage using PELCO® Pro CA44 Tissue Adhesive (TED PELLA INC., Prod. No. 10033) and embedded in 8% UltraPure™ LMP Agarose (Invitrogen, Cat. No. 16520-100). 100 µm thick, longitudinal root tissue sections were cut from root tips using a Vibratome Series 1500 (The Vibratome Company, discontinued). Subsequent sample fixation, dehydration, permeabilization, protease digestion, probe hybridization and amplification was performed as described previously (*71*).

After hybridization and washes, roots with were left in ClearSee [10% Xylitol (Sigma, CAS-No: 87-99-0), 15% Sodium deoxycholate (Sigma, CAS-no: 302-95-4), 25% Urea (Sigma, CAS-No: 57-13-6)] (*50*) overnight at 4 degrees Celsius and then stained with 0.22% Calcofluor-white (Fluorescent Brightener 28, Santa Cruz Biotechnology sc-218504) to visualize tissue layers by staining cell walls. Samples were mounted on glass slides and imaged with a SP8 Leica Confocal Microscope (Leica Microsystems), using a 20X oil immersion objective. For Calcofluor-white stain, excitation was done at 405 nm and collection at 425-475 nm. For imaging Alexa 647-coupled amplifiers, excitation was performed at 640 nm and collected at 650-675 nm. Leica LAS X Navigator software was used to collect and merge tiled images.

Imaging of auxin reporter DII-VENUS-NLS

Seeds for DII-VENUS-NLS (*72*) in maize inbred B73 were obtained from Carolyn Rasmussen (University of California, Riverside) and propagated by backcrossing into inbred line B73. For the imaging of hydropatterning induced auxin gradients in root tips, DII-VENUS-NLS plants were grown in the hydropatterning assay as described above. After 2 to 3 days in the hydropatterning assay, 3 cm root tip sections were harvested and immediately fixed in 4 % paraformaldehyde (Electron Microscopy Sciences, CAS #30525-89-4) in 1X phosphate buffered saline (PBS) (VWR International, Item No. MRGF-6235). To mark the air-side of the root, a small piece of mesh from the hydropatterning assay was glued (TED PELLA INC., Prod. No. 10033) to this side. Root tip sections were fixed for 1 hour at room temperature under vacuum. After fixation, root tips were washed with 1 X PBS and cut into 100 µm thick longitudinal sections using a Vibratome Series 1500 (The Vibratome Company, discontinued). Root tip orientation was marked by a 45˚ cut into the root according to the mesh orientation. After sectioning, root tip sections were cleared in ClearSee [10% Xylitol (Sigma, CAS-No: 87-99-0), 15% Sodium deoxycholate (Sigma, CAS-no: 302-95-4), 25% Urea (Sigma, CAS-No: 57-13-6)] (48) overnight at room temperature and then stained with 0.22% Calcofluor-white (Fluorescent Brightener 28, Santa Cruz Biotechnology sc-218504) to visualize tissue layers by staining cell walls. Samples were mounted on glass slides and imaged with a SP8 Leica Confocal Microscope (Leica Microsystems), using a 20X oil immersion objective. For Calcofluor-white stain, excitation was done at 405 nm and collection at 431-460 nm. DII-VENUS-NLS excitation was

done at 515 nM and collected at 520-550 nm. Leica LAS X Navigator software was used to collect and merge tiled images.
Quantification of the nuclear localized DII-VENUS signal was done using the Aivia software (Leica). Briefly, a region of interest was created for the first 2.6 mm of the root tip. A "Pixel classifier" was trained to recognize nuclei. Using the recipe "Cell Count - Cellpose" nuclei were segmented from the image with the help of "Pixel classifier" overlay. Nuclei that were closer than 15 µm from the outside edge of the root were removed to avoid detecting signal artifacts that could be created by the glue around the root sections. Nuclei were split into contact- and air-side groups by dividing the root section in the center (using the information from the 45˚ cut). For each nucleus maximum signal intensity was obtained and mean values were calculated for contact- and air-side nuclei each. Paired comparisons were done using the Student's t-test on *n* = 7 roots.

<u>Arabidopsis hydropatterning assay</u>

Phenotyping: Hydropatterning plate assays were performed to identify mutants that showed different hydropatterning behavior compared to Col-0 wild-type (fig. S8A). Seeds were surface sterilized for 10 min. using a 25% (v/v) bleach and 0.1% Tween solution and then washed four times with sterile water. Sterilized seeds were placed on gellan gum plates with 4.3g/L Murashige & Skoog Salts (Caisson Labs, Ref. No. MSP01-50LT), 1% sucrose (Sigma-Aldrich, CAS No. 57-50-1), 0.05% MES Hydrate (Sigma-Aldrich, CAS No. 1266615-59-1), and 0.7% Gelzan (Sigma-Aldrich, CAS No. 71010-52-1) and stratified for 72 hours at 4 ℃ in the dark. Each plate contained five Col-0 wild-type seeds and five mutant seeds that were sown about 1 cm from the top. After stratification, plates were then wrapped in parafilm (Fisher Scientific, Cat. No. 13-374-10) with a single layer of micropore tape (VWR International, Cat. No. 56222-182) at the top and placed on a rack to sit vertically in the growth cabinet (Percival Scientific, Model: CU-36L4). Plants were grown under long day conditions (16 hours light/ 8 hours dark, 22 ℃, 37% relative humidity) for 11 days. After 11 days of growth, lateral roots were quantified on the contact- and air-side using a S9 E StereoZoom Microscope (Leica, PN: 10 450 814). Lateral roots were classified as "contact-side" when they emerged from the primary root in directions growing on or into the media. All other lateral roots were classified as "air-side". Subsequently, plates with seedlings were imaged using an Epson Perfection V800 Photo Scanner (Seiko Epson Corporation, Model B11B223201).

Image analysis: Root length was measured using a custom built macro *Arabidopsis_RootTrace.ijm* (*41*) in ImageJ/Fiji (*46*).

Data analysis and visualization: Lateral root counts from the manual quantification via microscopy and root length measurements from images were imported into R version 4.2.1 (*47*). Data analysis and visualization were performed with the help of the *tidyverse* R package (*48*) and the following custom scripts: *ArabidopsisHydropatterning_MutantScreen.Rmd*, *ArabidopsisHydropatterning_EthylenePathway.Rmd*, and *ArabidopsisHydropatterning_Pharma.Rmd* (*41*). Each plate was treated as a biological replicate, since individual seedlings make only few air-side lateral roots each leading to a large uncertainty when estimating the true frequency of air-side lateral roots for a genotype. For contact- and air-side lateral root density, contact- and air-side lateral root counts were summed up for all Col-0 wild-type and all mutant seedlings per plate and divided by the cumulative root length. Root length was measured from the topmost to the bottommost lateral root that was counted. Likewise, percent air-side lateral roots were calculated as the sum of all air-side lateral roots per

genotype/plate divided by the total sum of all lateral roots per genotype/plate and then multiplied by 100. To compare the effects of mutants relative to Col-0 wild-type paired Student's t-tests were used treating each plate as a statistical unit. In cases of comparing treatment effects, plates were still treated as a statistical unit, but non-paired Student's t-tests were used for comparisons. For comparisons of multiple groups ANOVA with post-hoc Tukey HSD Test were performed.

Pharmacological treatment - Arabidopsis

AIB and ACC treatments: Gellan gum plates for the Arabidopsis hydropatterning assay (see above) were prepared with the addition of 2-aminoisobutyric acid (AIB) (Fisher Scientific, Cat. AAA1302114) and/or 1-aminocyclopropane-1-carboxylate (ACC) (PhytoTech Lab, Cat. A1180). Stock solutions (1 M AIB, 10 mM ACC) were prepared in water, filter-sterilized, and added to the media just before pouring the plates. The hydropatterning assay was performed as described above.

Ethylene treatment: Plates with seeds for the hydropatterning assay were prepared as described above. Two days after germination, primary root positions were marked on plates to denote the start of the treatment. Plates were transferred into 2.6 L air-tight storage containers (OXO, Model: 11233600) which were modified to include a rubber stopper for ethylene injections and placed upside down. Container gaskets were lubricated for a better seal with high vacuum grease (Dow Corning®, CAS #7631-86-9). Ethylene (Linde Part #: AI EY1C-A3) was injected to a final concentration of 0.2 ppm. In each control container one WiseFresh-Ethylene Absorber package (WiseSorbent Technology, SKU #3ESTY001A) was included to remove any possible contaminant ethylene. Plants were grown in the containers under long day conditions (16 hours light/ 8 hours dark, 22 ℃, 37% relative humidity) for 11 days. All containers were opened once daily and ethylene was freshly injected after resealing. Phenotyping was conducted as described above.

Pharmacological treatment - maize

AIB treatment: Two tanks (n = 20 seedlings/tank) with the custom maize hydropatterning assay were prepared as described above. Each tank was filled with 6 L water. One tank was treated with a mock solution while the other tank was treated with a 1 M solution of 2-aminoisobutyric acid (AIB) (Fisher Scientific, Cat. AAA1302114) to a final concentration of 10 mM. Seeds of the strong hydropatterning maize inbred line 33-16 were pre-germinated on plates that were treated either with a mock solution or with 10 mM AIB. Plants were grown, harvested and analyzed as described above.

Ethylene quantification

Maize root tips were collected for ethylene measurements immediately after harvesting maize plants from the hydropatterning assay. For this, 1cm long root tip sections of four plants were pooled and rolled up in a 1 x 3 cm strip of pre-moistened tissue paper and placed into 2 mL Screw Glass Vials (Thermo Scientific, Cat. No. 6PSV9-1P) filled with 200 µL of water. Ethylene was allowed to accumulate for 29 hours before measurements using a gas chromatograph (Shimadzu, Model GC-8A). From each vial, 1 mL of sample was injected into the gas chromatograph. Ethylene production was normalized by the fresh weight of the root samples.

Rice hydropatterning assay

Rice (*Oryza sativa*) dehusked seeds of wild-type Nipponbare and *osein2* mutant were surface sterilized for 4 min. in 100% bleach (CaOCl2 concentration 20%) and then washed 5 times in

autoclaved, deionized water. Seeds were sown on plates containing ½ strength MS and 1% BD Difco Agar (Fisher Scientific) at a pH of 5.8. Three days post germination, seedlings were transferred to fresh plates and their root tips marked with a dot. Plants were grown in a Conviron growth chamber at 28 ˚C, 70% relative humidity and 150 μmol m^{-2} s^{-1} light intensity at 16-8 hour day-night cycle. All plates were placed in a vertical orientation with an inclination of ~80° to allow the roots to grow along the surface of ½ MS agar. All hydropatterning measurements were taken from the root growth after transfer (starting from the dot) as primary root orientation might get slightly altered during the transfer process. Four days after transfer, the plates were imaged and scored for lateral root hydropatterning in a manner similar to that used with Arabidopsis.

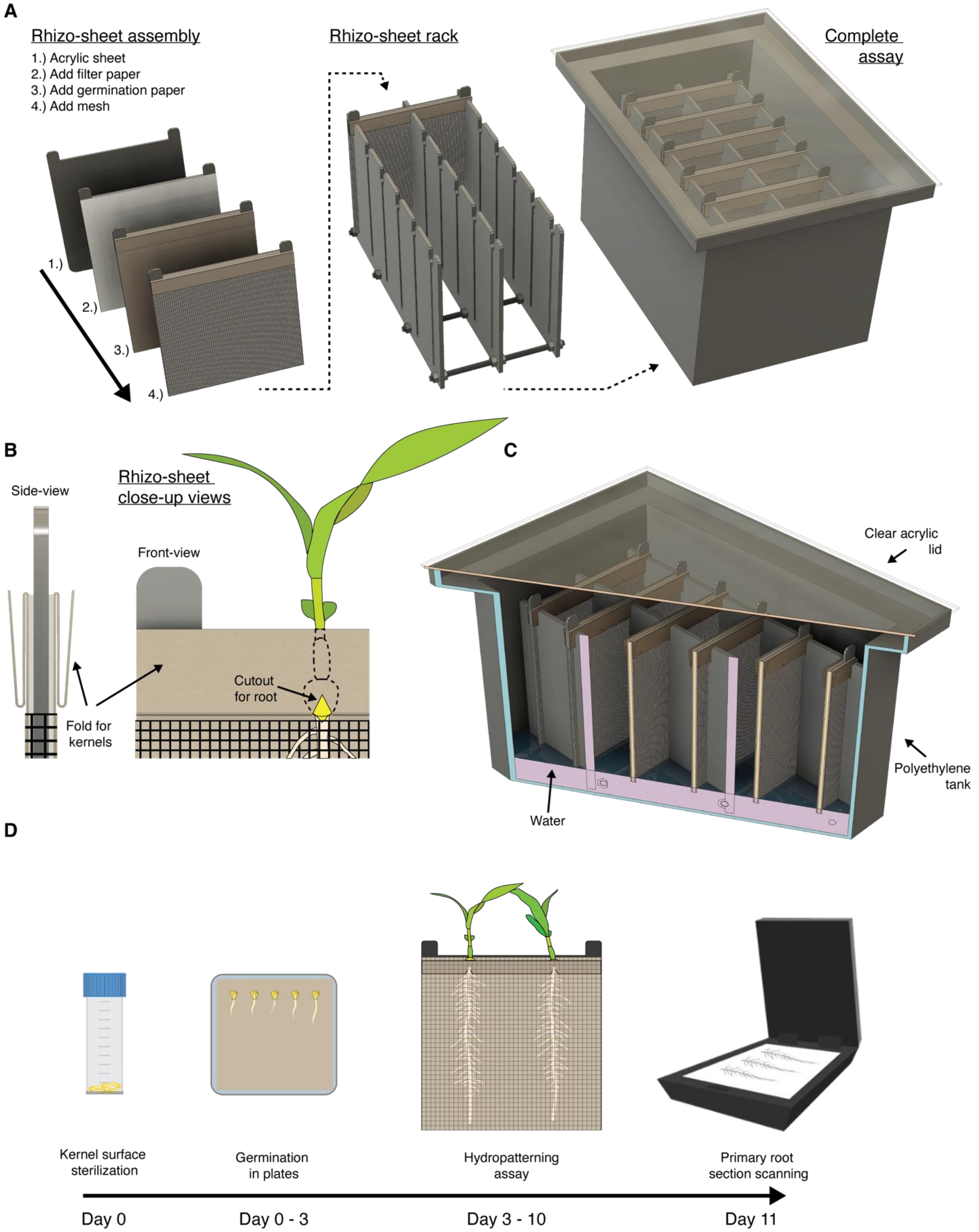

fig. S1. Design assembly, and workflow of our custom-built assay to characterize hydropatterning in *Zea mays* (maize). (A) Assembly of the hydropatterning assay: (I) Rhizo-sheets were made from an acrylic panel that was covered on both sides with a layer of filter

paper and pre-folded germination paper. The assembly was covered tightly by a layer of window mesh that had been fashioned into a pouch. (II) Rhizo-sheet racks were assembled from laser cut acrylic panels, PVC rods, and PVC nuts and designed to hold six rhizo-sheets each. (III) Polyethylene tanks with clear acrylic lids were used to house the rhizo-sheet racks with rhizo-sheets when running the hydropatterning assay. **(B)** Close-up view of the folded germination paper at the top of the rhizo-sheets. The side-view shows the folded channel to hold germinated maize kernels. The front view shows the triangular cutout that was introduced to let the primary root exit the channel and grow beneath the window mesh along the moist germination paper. **(C)** Cross-section view of the hydropatterning assay showing a 1.5” deep layer of water at the bottom of the tank which kept the rhizo-sheets moist throughout the duration of the experiment. **(D)** Workflow of the hydropatterning assay in maize: (Day 0) Maize kernels were surface sterilized (Day 0 - 3) Sterilized kernels were transferred to plates with moist germination paper and incubated for three days. (Day 3 - 10) Germinated seedlings were immediately transferred to the hydropatterning assay and grown for seven days. (Day 11) 12 cm long primary root sections were cut from the hydropatterning assay and lateral root branch patterns of the contact-and air-side were recorded using a flat-bed scanner.

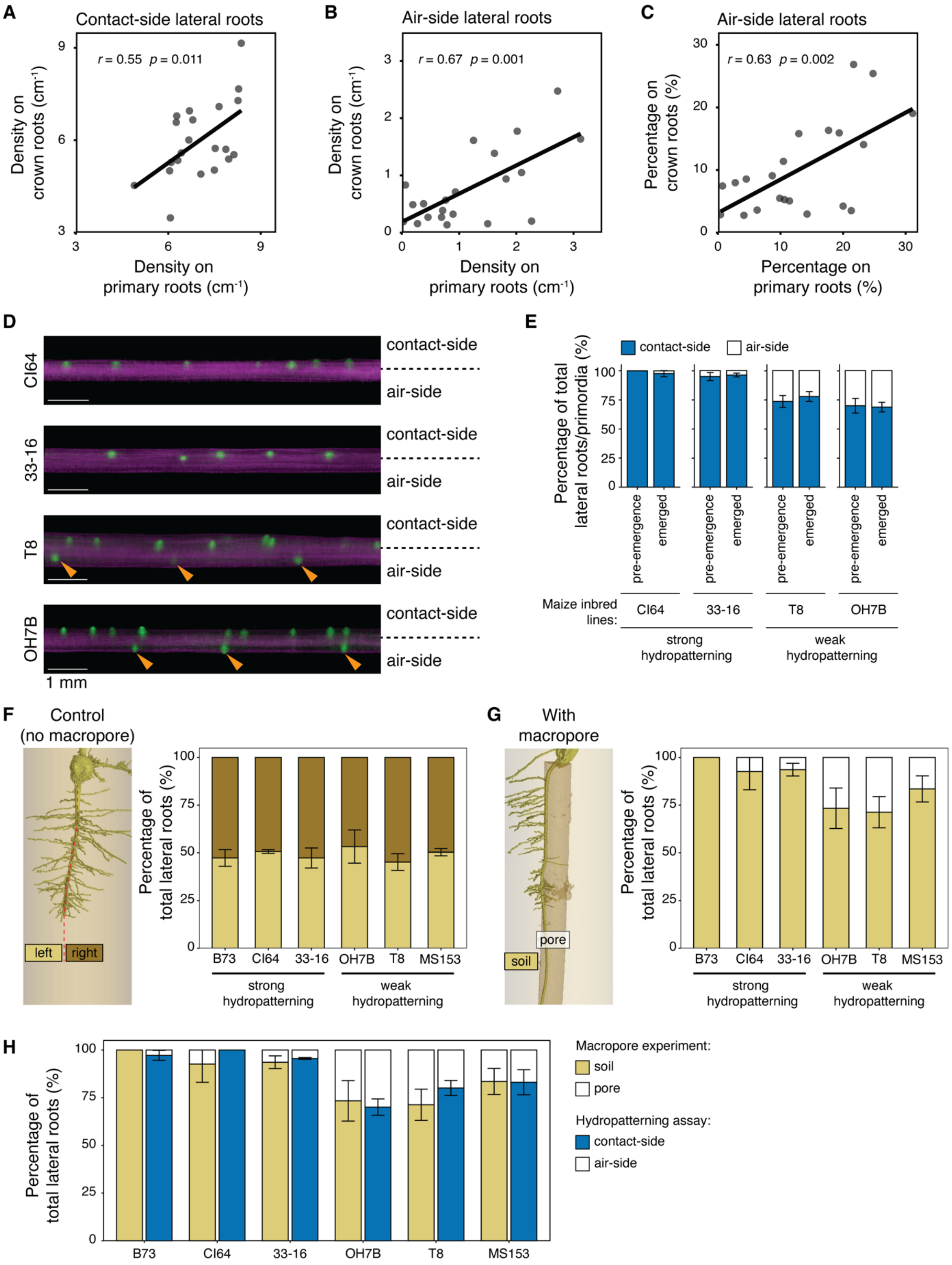

fig. S2. Primary and crown root comparison, microscopy, and X-ray Computed Tomography of hydropatterning in *Zea mays* (maize). (A - C) Comparisons of (A) contact-

side lateral root density, (B) air-side lateral root density, and (C) percent air-side lateral roots on primary and crown roots of $n = 21$ maize inbred lines. Points show the inbred line mean value of 1-4 plant replicates. Black solid line is the linear regression line. Pearson's correlation (r) and p-value are shown. **(D and E)** Fluorescence microscopy and quantification of pre-emergence lateral root primordia in four maize inbred lines (CI64, 33-16, T8, OH7B). Confocal images (D) show the early mature zone of primary roots for seedlings that were grown in the hydropatterning assay. Lateral root primordia are marked by SYBR GREEN (green) and counterstained with Basic Fuchsin (magenta). Air-side lateral root primordia are indicated by orange triangles. White scale bar: 1 mm. Quantitative comparisons (E) of pre-emergence lateral root primordia, assessed via microscopy, and post-emergence outgrown lateral roots, counted on images from the hydropatterning assay. $n = 5 - 10$ seedlings/inbred line. **(F - H)** Quantification of lateral root patterning on primary roots in soil via X-ray Computed Tomography for three strong (B73, CI64, 33-16) and three weak (OH7B, T8, MS153) hydropatterning maize inbred lines and comparison to lateral root patterning in the hydropatterning assay. (F) Control experiment with roots fully surrounded by soil. Lateral root emergence points were quantified left and right of the primary root axis. $n = 2 - 3$ seedling replicates/inbred line and n = 36 - 110 lateral roots/replicate. (G) Primary roots grown through an air-filled macropore in soil. Lateral root emergence points were quantified towards the soil and towards the air-filled macropore. $n = 2 - 4$ seedling replicates/inbred line and $n = 13 - 42$ lateral roots/replicate. (H) Comparisons of lateral root patterning in the air-filled macropore to lateral root patterning in the hydropatterning assay for the same inbred lines. Hydropatterning assay: $n = 3$ replicates/inbred line. Bar graphs: mean +/- SEM.

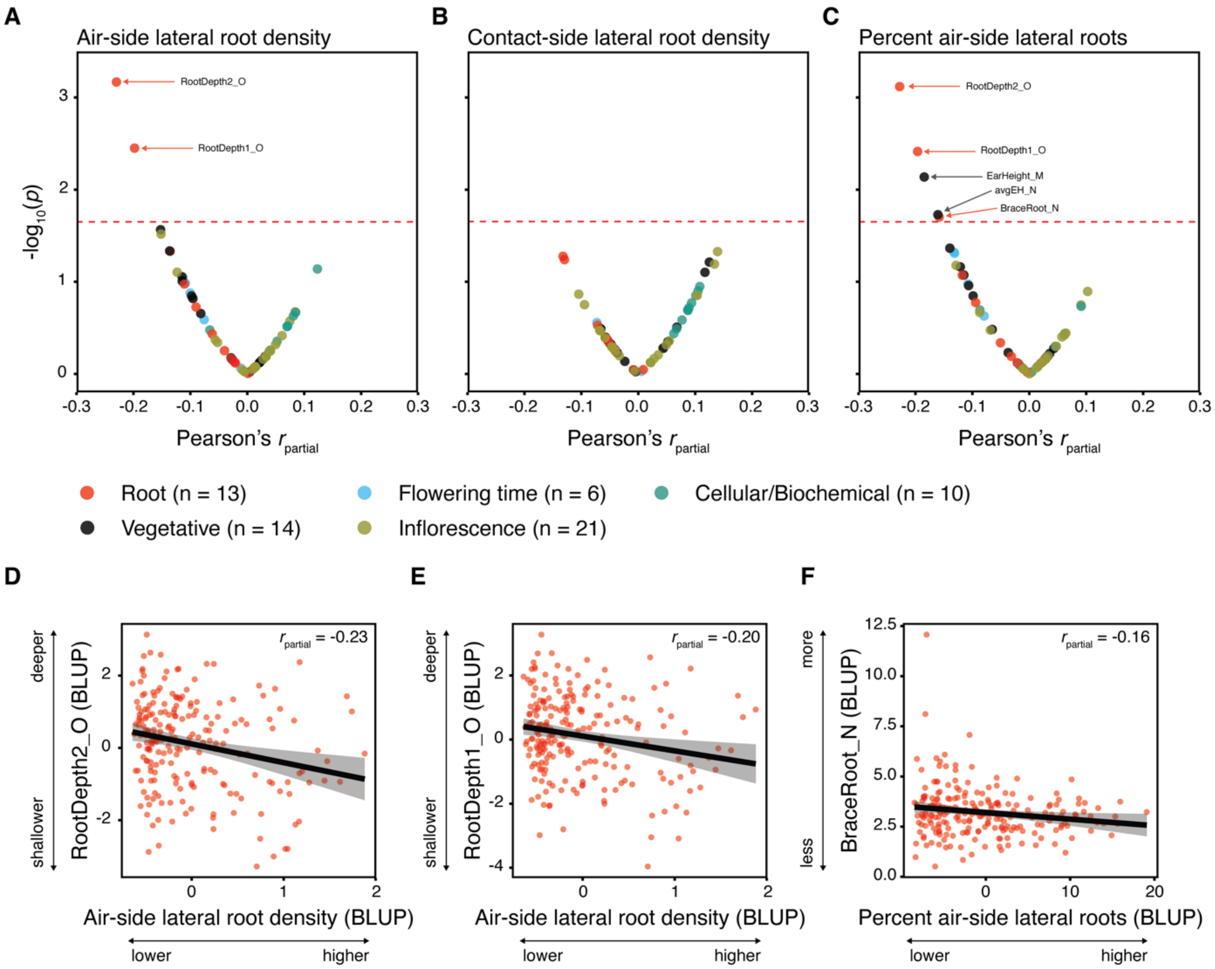

fig. S3. Correlation analysis of hydropatterning traits with phenotypic traits of field-grown maize plants. (A - C) Volcano plots for correlations between (A) air-side and (B) contact-side lateral root density, and (C) percent air-side lateral roots and 67 quantitative phenotypic trait sets collected from field-grown maize (*11*). Pearson's correlation coefficients were adjusted for population structure ($r_{partial}$). Data points are colored by trait group. Pairwise-complete genotypes (inbred lines) ranged from 195 - 230 per trait set. Red dashed line marks the FDR-corrected threshold at p = 0.05. **(D - F)** Partial residuals plot showing the correlations between Best Linear Unbiased Predictions (BLUPs) of (D) air-side lateral root density and RootDepth2_O, (E) air-side lateral root density and RootDepth1_O, and (F) percent air-side lateral roots and BraceRoot_N. RootDepth1_O and RootDepth2_O are measures of root crown depth obtained from excavated root crowns (*12*); n = 218 pairwise-complete genotypes (inbred lines). BraceRoot_N is a measure of the number of nodes with brace roots (*13*); n = 219 pairwise-complete genotypes (inbred lines).Population structure was added as a covariate. Black solid lines (linear regression lines), standard error (gray shaded area).

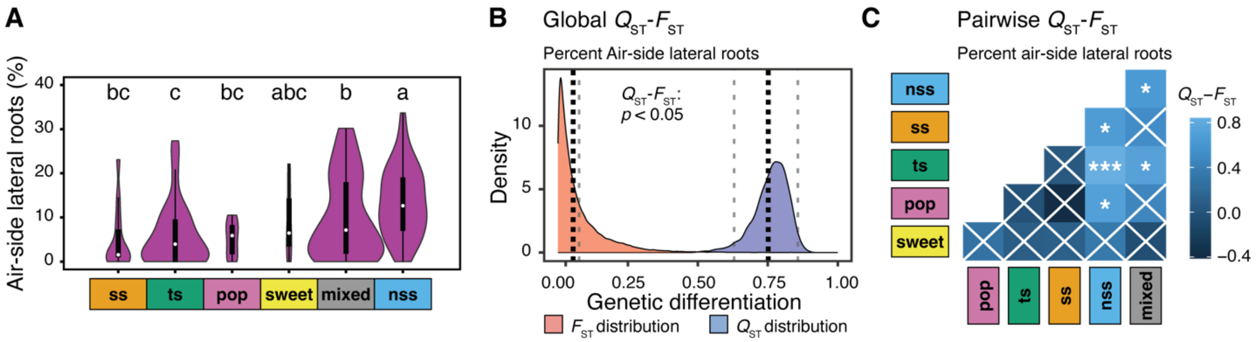

fig. S4. Phenotypic variation and signatures of selection for percent air-side lateral roots. (**A**) Comparisons of percent air-side lateral roots between subpopulations (ss = stiff stalk, ts = tropical and subtropical, pop = popcorn, sweet = sweet corn, mixed = mixed, nss = non-stiff stalk). Violin plot areas were adjusted by number of inbred lines in each subpopulation (n_{ss} = 14, n_{ts} = 53, n_{pop} = 9, n_{sweet} = 6, n_{mixed} = 88, n_{nss} = 63). Letters denote significant differences between subpopulations ($p \leq 0.05$, Kruskal–Wallis and Dunn's post hoc tests); n.s., no significant differences. (**B**) Population-wide comparison of F_{ST} (fixation index) and Q_{ST} (genetic differentiation regarding percent air-side lateral roots). Black dotted lines denote means; gray dashed lines denote confidence intervals. (**C**) Subpopulation pairwise Q_{ST} - F_{ST} comparisons. Asterisks denote significant differences between Q_{ST} and F_{ST} (* $p \leq 0.05$, ** $p \leq 0.01$, *** $p \leq 0.001$). White crosses: not significant.

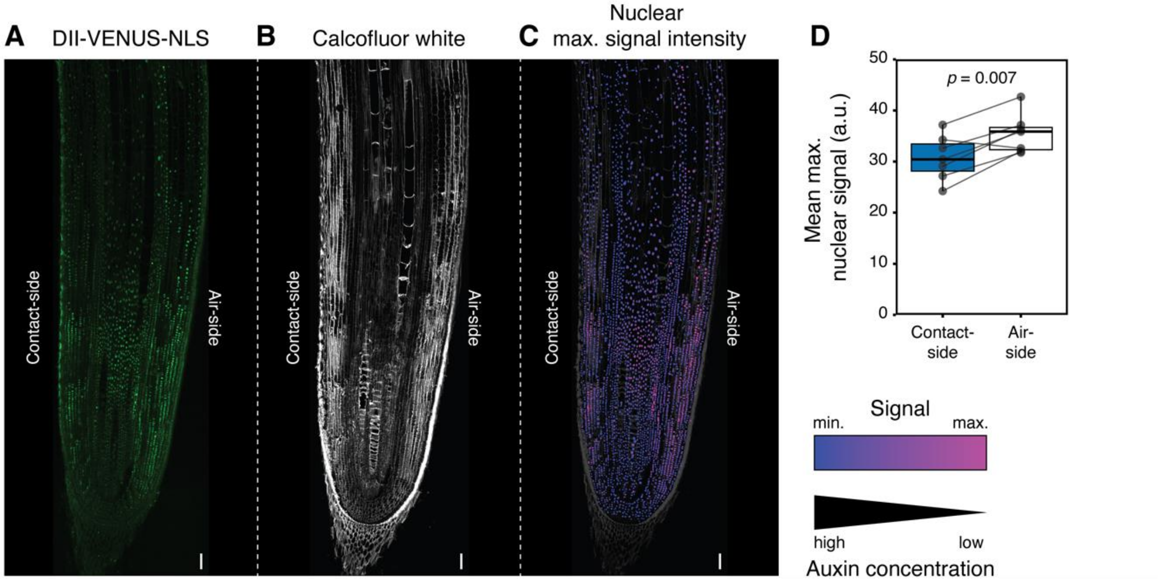

fig. S5. Auxin bias between contact- and air-side of *Zea mays* (maize) primary root tips. (A) Confocal image of a longitudinal primary root tip section from a maize plant grown in the hydropatterning assay and expressing the auxin reporter DII-VENUS-NLS. Contact-side (left) and air-side (right) are annotated. **(B)** Same root section showing cell walls stained with calcofluor for anatomy. **(C)** Image of computationally dissected nuclei showing their maximum intensity value (gradient from blue to magenta) quantified from the DII-VENUS-NLS image and superimposed onto the root section stained with calcofluor white (gray). **(D)** Quantification of DII auxin reporter signal in longitudinal sections of primary root tips from n = 7 plants that were grown in the hydropatterning assay. Global means of maximum intensity values from all nuclei on the contact- and air-side were calculated and compared using the Student's t-test. White scale bar = 100 μm.

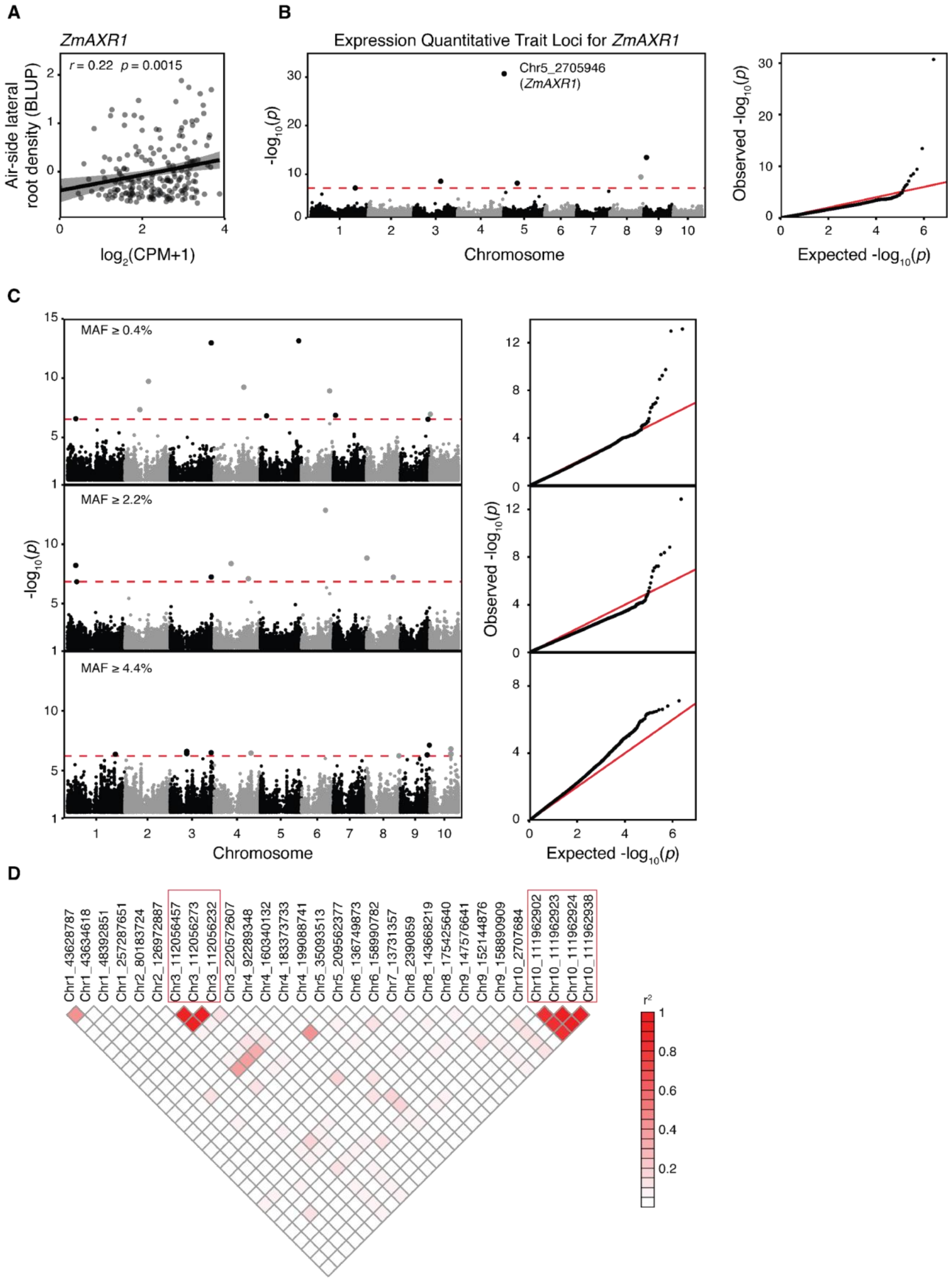
A
ZmAXR1
r = 0.22 p = 0.0015
Air-side lateral root density (BLUP)
log2(CPM+1)
B
Expression Quantitative Trait Loci for ZmAXR1
Chr5_2705946
(ZmAXR1)
-log10(p)
Chromosome
Observed -log10(p)
Expected -log10(p)
C
MAF ≥ 0.4%
MAF ≥ 2.2%
MAF ≥ 4.4%
-log10(p)
Chromosome
Observed -log10(p)
Expected -log10(p)
D
Chr1_43628787
Chr1_43634618
Chr1_48392851
Chr1_257287651
Chr2_80183724
Chr2_126972887
Chr3_112056457
Chr3_112056273
Chr3_112056232
Chr3_220572607
Chr4_92289348
Chr4_160340132
Chr4_183373733
Chr4_199088741
Chr5_35093513
Chr5_209562377
Chr6_136749873
Chr6_158990782
Chr7_13731357
Chr8_2390859
Chr8_143668219
Chr8_175425640
Chr9_147576641
Chr9_152144876
Chr9_158890909
Chr10_2707684
Chr10_111962902
Chr10_111962923
Chr10_111962924
Chr10_111962938
r²

fig. S6. Genome and Transcriptome Wide Association Studies for hydropatterning in *Zea mays* (maize). (**A**) Association between gene expression of *ZmAXR1* (*Zm00001eb211770*) in seedling roots of n = 206 maize inbred lines (*19*) and Best Linear Unbiased Predictors (BLUPs) for air-side lateral rotor density. Black solid line and gray shaded areas show the linear regression line and standard error. (**B**) Manhattan and associated quantile-quantile plot for the mapping of expression Quantitative Trait Loci for *ZmAXR1*. The red horizontal dashed line marks the FDR adjusted significance threshold at p = 0.05. (**C**) Manhattan and associated quantile-quantile plots for the Genome Wide Association Study on air-side lateral root density using a total of 1.2 million high-density SNPs (*66*). The three stacked plots show GWAS results for runs at three Minor Allele Frequency (MAF) cutoffs ≥ 0.4%, ≥ 2.2%, ≥ 4.4% which were used to select Trait Associated SNPs (TAS). The red horizontal dashed lines mark the FDR adjusted threshold at p = 0.05. (**D**) Linkage Disequilibrium matrix of squared correlations (r^2) between all TAS detected by GWAS. Red boxes mark groups of adjacent TAS that are highly (r^2 ≥ 0.8) correlated.

1

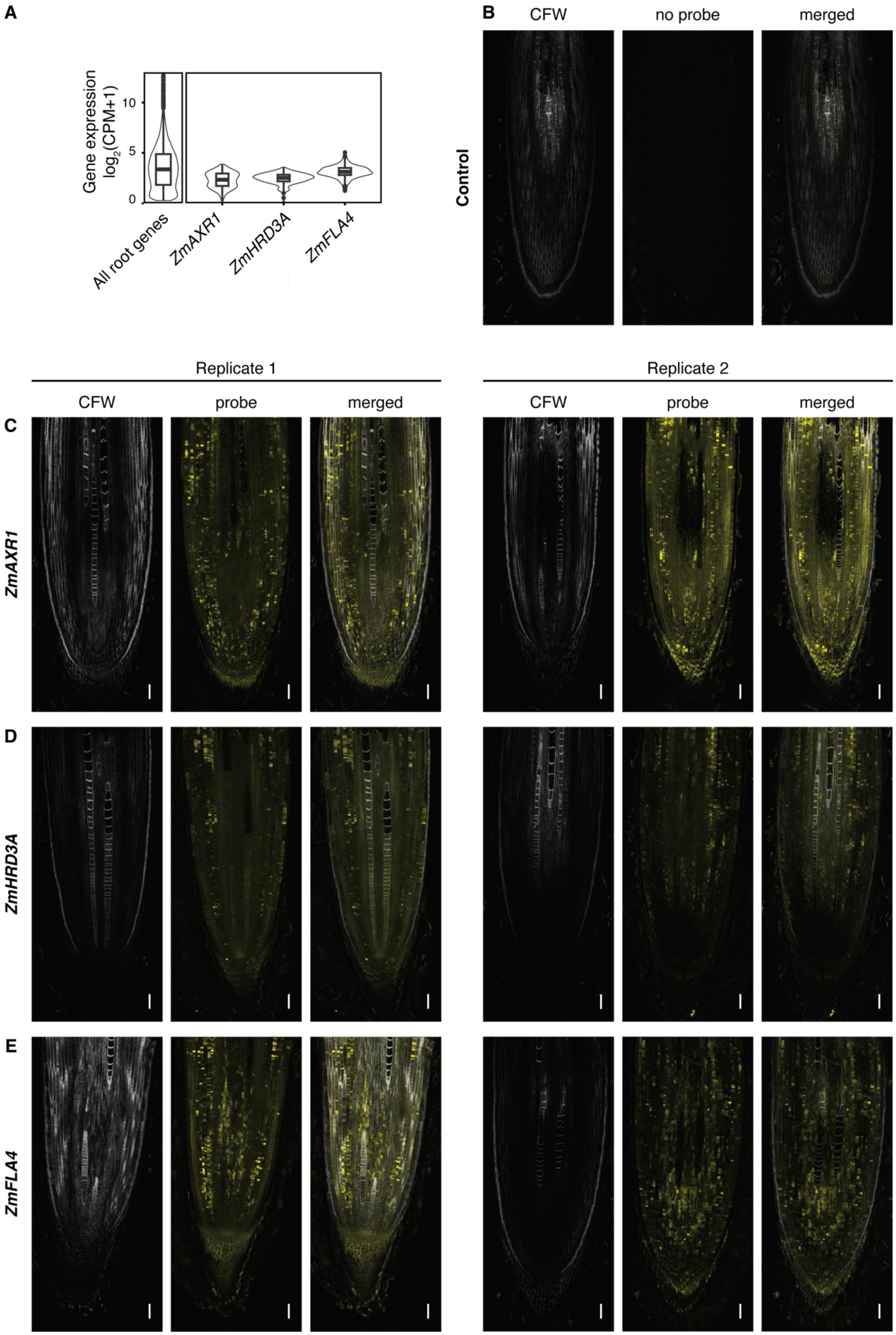

A
Gene expression log$_2$(CPM+1)
0
5
10
All root genes
ZmAXR1
ZmHRD3A
ZmFLA4
B
CFW
no probe
merged
Control
Replicate 1
Replicate 2
CFW
probe
merged
CFW
probe
merged
C
ZmAXR1
D
ZmHRD3A
E
ZmFLA4

fig. S7. Gene expression analysis and Hybridization Chain Reaction in *Zea mays* (maize). (**A**) Gene expression of *ZmAXR1* (*Zm00001eb21177*0), *ZmHRD3A* (*Zm00001eb398230*), and *ZmFLA4* (*Zm00001eb367960*) in root tips of maize seedlings (*19*). Violin and integrated boxplots show the expression across 206 maize inbreds. “All root genes” shows the distribution of the median expression for all root expressed genes (n = 21606) across the 206 maize inbred lines. (**B - E**) Image from Hybridization Chain Reaction experiments separated into a channel for Calcofluor White (CFW), a channel for the probe, and a combined channel (merge). (B) Control experiment with no Hybridization Chain Reaction probes added. (C - E) Two independent Hybridization Chain Reaction experiments with probes against (C) *ZmAXR1*, (D) *ZmHRD3A*, and (E) *ZmFLA4*. White scale bars = 100 μM.

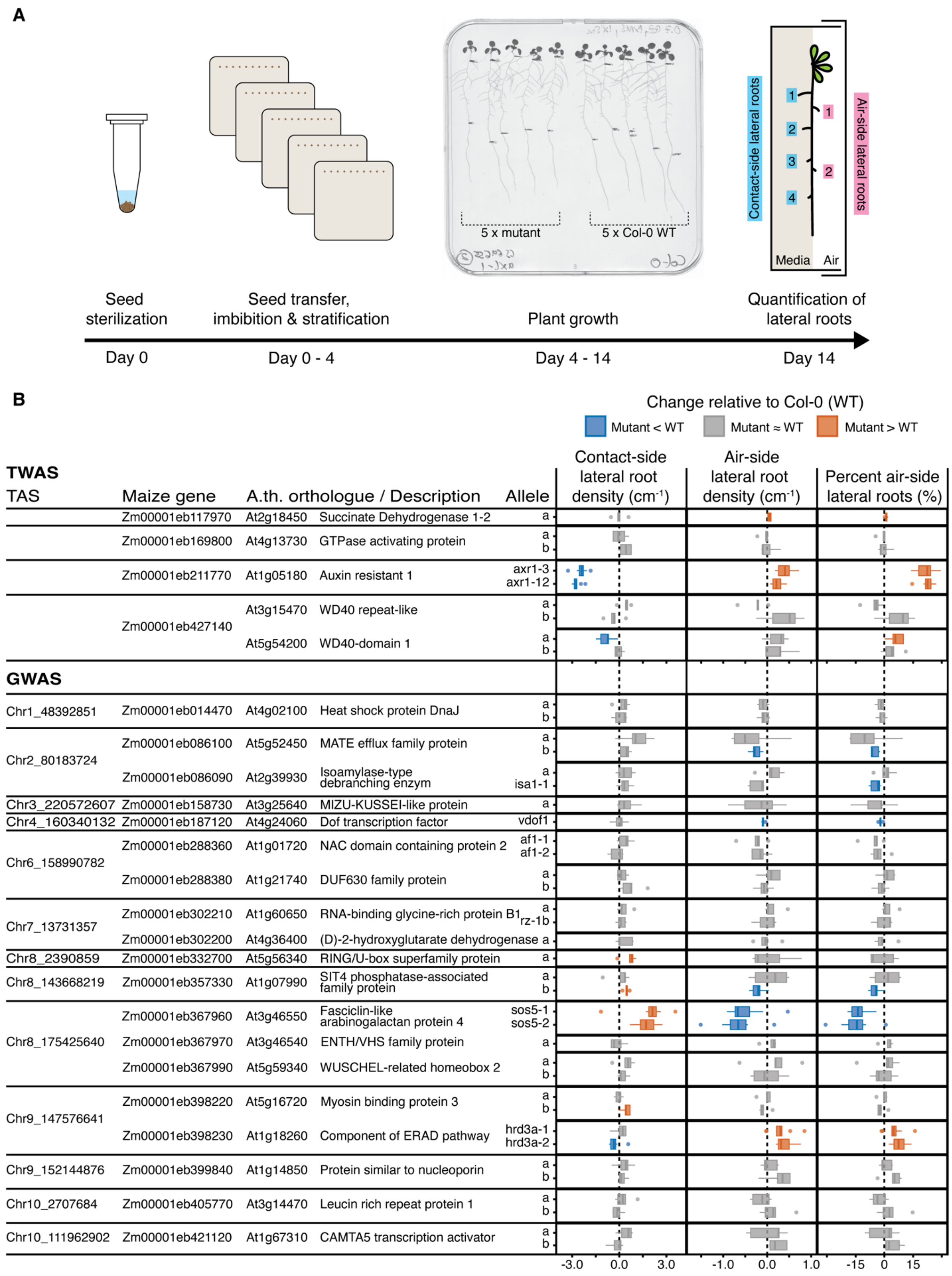

fig. S8. Screening of orthologous genes in Arabidopsis for GWAS and TWAS validation.
(A) Workflow of the hydropatterning assay in Arabidopsis: (Day 0) Arabidopsis seeds are

surface sterilized (Day 0 - 4) Sterilized seeds are transferred to gel-plates and stratified for three days at 4 ˚C (Day 4 - 14) Seedlings on plates are grown inside a growth chamber for 11 days (Day 14) Lateral roots are quantified using a stereo microscope and plates are imaged using a flatbed scanner for measurements of root length. (**B**) Overview of changes in contact-side lateral root density, air-side lateral root density, and percent air-side lateral roots relative to Col-0 (WT) for 42 Arabidopsis mutant lines. TAS refers to landmark SNP relating to the maize genes identified in through GWAS. *n* = 5 - 10 plates/mutant (5 WT & 5 mutant plants/plate). Boxplot fill color indicates statistical differences (Paired Student's t-test, $p \leq 0.05$): significant decrease relative to WT (blue), significant increase relative to WT (orange), and no significant change (gray).

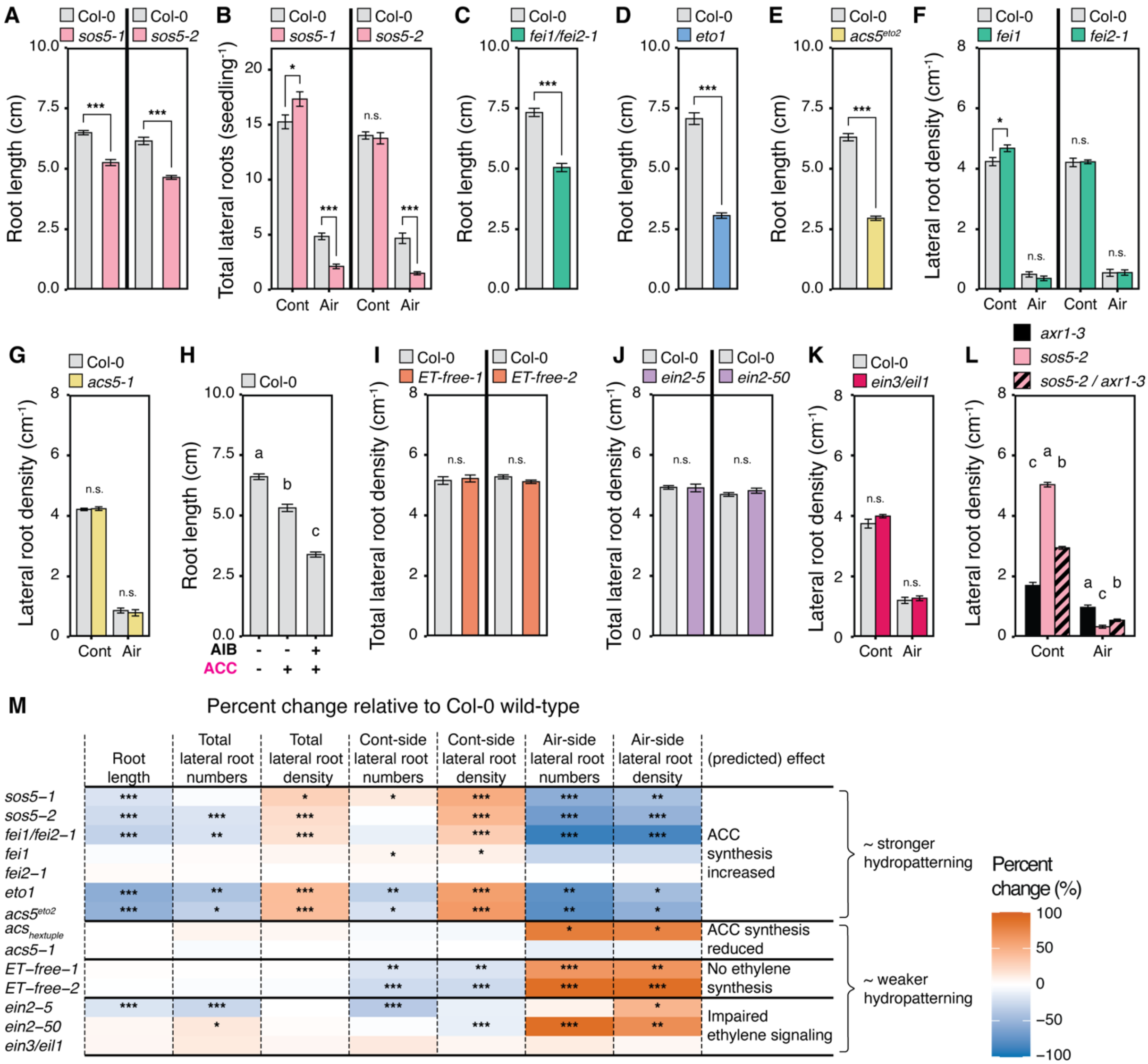

fig. S9. Additional comparisons of mutants and pharmacological treatments related to the ethylene pathway in Arabidopsis. (**A, and C - E**) Comparisons of total root length between Col-0 (gray) and (A) *sos5-1* & *sos5-2* (*n* = 10 plates each), (C) *fei1/fei2-1* (*n* = 6 plates), (D) *eto1* (*n* = 5 plates), and (E) *acs5*^{eto2} (*n* = 5 plates). (**B**) Comparisons of the total number of contact-side (Cont) and air-side (Air) lateral roots per seedling between Col-0 and *sos5-1* & *sos5-2* (*n* = 10 plates each). (**F and G**) Comparisons of contact-side (Cont) and air-side (Air) lateral root densities between Col-0 and (F) *fei1* & *fei2-1* (*n* = 6 plates each), and (G) *acs5-1* (*n* = 5 plates). (**H**) Comparisons of total root length between Col-0 treated with a (-) mock solution, (+) 0.05 mM ACC, or (+) 0.05 mM ACC + 5 mM AIB (*n* = 10 plates). (**I, J**) Comparisons of the total number of lateral roots per seedling (contact- and air-side combined) between Col-0 and (I) *ET-free-1* (*n* = 8 plates) & *ET-free-2* (*n* = 9 plates), and *ein2-5* & *ein2-50* (*n* = 10 plates each). (**K**) Comparisons of contact- and air-side lateral root densities between Col-0 and *ein3/eil*1(*n* = 10 plates each). (**L**) Comparisons of Cont and Air lateral root densities between *axr1-3*, *sos5-2*, and *sos5-2/axr1-3* (*n* = 10 plates each). (**M**) Overview of Arabidopsis mutant responses relative to Col-0 wild-type. Responses are shown as percent change calculated based on the mutant / wild-type mean value. General: Asterisks denote significant differences (Paired Student's t-test: * *p* ≤

0.05, ** $p \leq 0.01$, *** $p \leq 0.001$) when Col−0 and mutants were grown on the same plates (5 Col−0 & 5 mutants / plates). Different letters denote significant differences (Student's t−test: $p \leq 0.05$ FDR−adjusted) between treatments or mutant lines when treatments/mutants were on different plates (10 plants/plate). Bar graphs: mean +/− SEM. n.s., not significant.
